# Formation, Transformation, and Synergistic Interplay of Crystalline Orientation in Conjugated Polymers via Solution‐State Aggregation Control

**DOI:** 10.1002/advs.77363

**Published:** 2026-08-25

**Authors:** Hao Zheng, Lixin Li, Yanan Guo, Bingjie Wu, Xuebing Luo, Hao Zhan, Yongjie Dong, Ze‐Fan Yao, Juan Peng, Jian Pei

**Affiliations:** ^1^ State Key Laboratory of Molecular Engineering of Polymers Department of Macromolecular Science Fudan University Shanghai China; ^2^ Beijing National Laboratory for Molecular Sciences (BNLMS) Key Laboratory of Polymer Chemistry and Physics of Ministry of Education Center of Soft Matter Science and Engineering College of Chemistry and Molecular Engineering Peking University Beijing China

**Keywords:** cocrystal, crystalline orientation, donor–acceptor polymers, solution–state aggregation, structure‐property relationship

## Abstract

Controlling and achieving desired crystal orientations (i.e., edge‐on and face‐on) in donor–acceptor (D–A) conjugated polymers is pivotal to enhancing their optoelectronic performance. Here, four diketopyrrolopyrrole (DPP)‐based polymers with different donor units (DPP3T, DPPTTT, DPP4T, and DPP5T) are developed to investigate the formation, transformation, and synergistic interplay of crystallite orientation. Three strategies were employed to probe these three aspects: (i) drop‐casting from different solvents to investigate the orientation formation governed by their solution‐state aggregation, (ii) thermal and solvent vapor annealing to achieve reversible orientation transformation, and (iii) blending to realize the orientation interplay between two polymers. During drop‐casting, DPPs with increased donor lengths and enhanced solution‐state aggregation favor the formation of edge‐on over face‐on orientation, where these two orientations in DPP3T can be further reversibly switched by thermal and solvent vapor annealing. Remarkably, by blending DPP3T and DPP5T with weak and strong solution aggregation, respectively, DPP5T could strengthen solution‐aggregates of DPP3T and drive them to form edge‐on DPP3T/DPP5T cocrystals. The charge mobilities of these DPPs correlate well with their crystalline structures. This study establishes the connection among solution‐state aggregates, solid‐state crystalline orientations, and charge‐transport properties in conjugated copolymers, contributing a deep understanding of their crystalline behavior for applications in optoelectronic devices.

## Graphical abstract

Molecular engineering (i.e., tuning donors) and three film processing strategies (i.e., drop‐casting from different solvents, thermal and solvent vapor annealing, and blending) were synergistically utilized to tailor the solution‐state aggregation and thus the formation, transformation, and interplay of solid‐state crystalline orientation (face‐on and edge‐on) in a series of diketopyrrolopyrrole (DPP)‐based copolymers. Their different crystalline structures greatly impacted their charge mobilities for use in high‐performance organic field‐effect transistors.

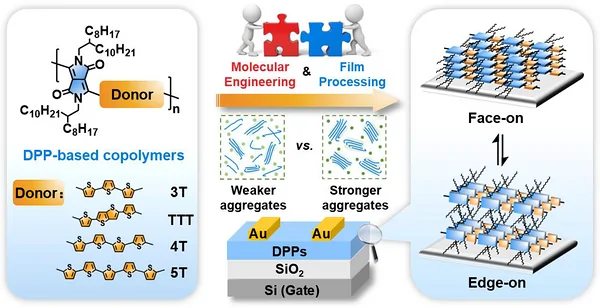

## 1 Introduction

Organic field‐effect transistors (OFETs) using conjugated polymers as the active layer have garnered extensive interest because of their easily modifiable chemical structures, mechanical flexibility, and solution processability [1, 2, 3, 4, 5]. The performance of OFETs (e.g., charge‐carrier mobility) is largely governed by the inherent chemical structures and multiscale solid‐state structures (e.g., crystallite orientation, polymorphs, and surface morphology) [6, 7, 8, 9, 10, 11]. Among the latter context, the orientation of crystallites (e.g., perpendicular vs. parallel to the substrate plane) is considered to play an important role in charge transport. An edge‐on crystallite orientation, where the conjugated backbone and π‐stacking direction lie in‐plane, is usually favorable for horizontal charge transport in OFETs [12, 13, 14]. A face‐on orientation, where the π‐stacking direction is vertical to the substrate plane, is advantageous for vertical charge transport in solar cells [15, 16]. In some cases, the coexistence of edge‐on and face‐on orientations, known as a bimodal structure, establishes three‐dimensional charge transport pathways, which significantly enhance carrier mobility [17, 18].

Solution‐state aggregation is regarded as a critical bridge between molecular structures of conjugated polymers and the solid‐state morphology and electronic property [19]. Compared to the widely studied poly(3‐alkylthiophene)s (P3ATs), the new generation D–A copolymers have more complex chemical structures, molecular interactions, and rigid chain characteristics, which made the previous understanding on the solution‐state aggregation behavior of P3ATs could not be directly extended to D–A copolymers [20, 21, 22]. Due to the memory effect, the aggregation behavior of conjugated polymers in the solution can be inherited to the solid state, thus impacting the device performance greatly [23, 24, 25, 26]. For example, we demonstrated that the solution‐state aggregates of a benzodifurandione‐based copolymer could be controlled by the solution temperature, and a high solution temperature could facilitate molecular motions to break the poorly packed aggregates in the solution and assist them to form ordered packed structures in the film, yielding up to two orders of magnitude in electron mobilities compared to those deposited at low solution temperatures [27]. Notably, due to the complicated dynamic behavior of solution aggregates and relatively limited characterization methods, comprehensive understanding on the processing‐affected solution‐state aggregation and how these aggregates impact the crystallite orientation in the film are still lacking. Moreover, conjugated polymer blends render collective properties over single constituent, which are often used in practical applications. However, research on controlling the solution aggregates and crystallite orientation of conjugated polymer blends is rather limited [28]. Clearly, the ability to reveal how the processing strategies influence the solution‐state aggregation and thus the crystallite orientation (i.e., face‐on and edge‐on) in the solid state is crucial to expand the efficient means of obtaining the desired crystallite orientation in conjugated polymers for high‐performance optoelectronics.

Among various conjugated polymers, diketopyrrolopyrrole (DPP)‐based copolymers have attracted much interest because of their excellent hole mobility [29, 30, 31, 32, 33]. Here, we present a systematic study on the formation, transformation, and synergistic interplay of crystallite orientations (face‐on and edge‐on) in a series of DPP‐based copolymers with different electron donors (DPP3T, DPPTTT, DPP4T, and DPP5T, where the donor units are 3T, TTT, 4T, and 5T, respectively), and establish the correlations between different crystalline structures and charge‐transport properties. Specifically, after drop‐casting (1st processing strategy) from chloroform (CHCl_3_), DPP3T displays a face‐on orientation in the film, whereas DPPTTT, DPP4T, and DPP5T exhibit an edge‐on orientation. When these DPPs are drop‐cast from o‐dichlorobenzene (ODCB), all of them exhibit an edge‐on orientation. The crystallite orientations of DPPs in the film are greatly influenced by the degree of their solution‐state aggregation. Intriguingly, the face‐on and edge‐on orientations in DPP3T can be reversibly transformed by alternating thermal and solvent vapor annealing (2nd processing strategy) driven by the mismatch between the thermodynamically stable states in the CHCl_3_ solution and in the thin film. Furthermore, when DPP3T and DPP5T (exhibiting weak and strong aggregation in CHCl_3_, respectively) are blended (3rd processing strategy) at certain weight ratios, the co‐aggregation between DPP5T and DPP3T in the solution enhances the aggregation of DPP3T itself and induces DPP3T transform from face‐on to edge‐on orientation; this is accompanied by the cocrystallization between DPP3T and DPP5T. Finally, these different crystalline structures of the DPP films are closely correlated with the OFET device performance, establishing clear structure–charge transport property relationships. This study comprehensively scrutinizes the solution‐state aggregation of DPP‐based copolymers via the concerted intrinsic molecular engineering and extrinsic processing strategies and how these solution aggregates influence the crystal orientation in the solid state, which provides a deep fundamental understanding on the crystalline behavior of D–A copolymers for high‐performance optoelectronic devices.

## 2 Results and Discussion

### 2.1 Formation of Crystal Orientation in DPP‐Based Copolymers via Drop‐Casting From Different Solvents (1st Processing Strategy)

**FIGURE 1:**
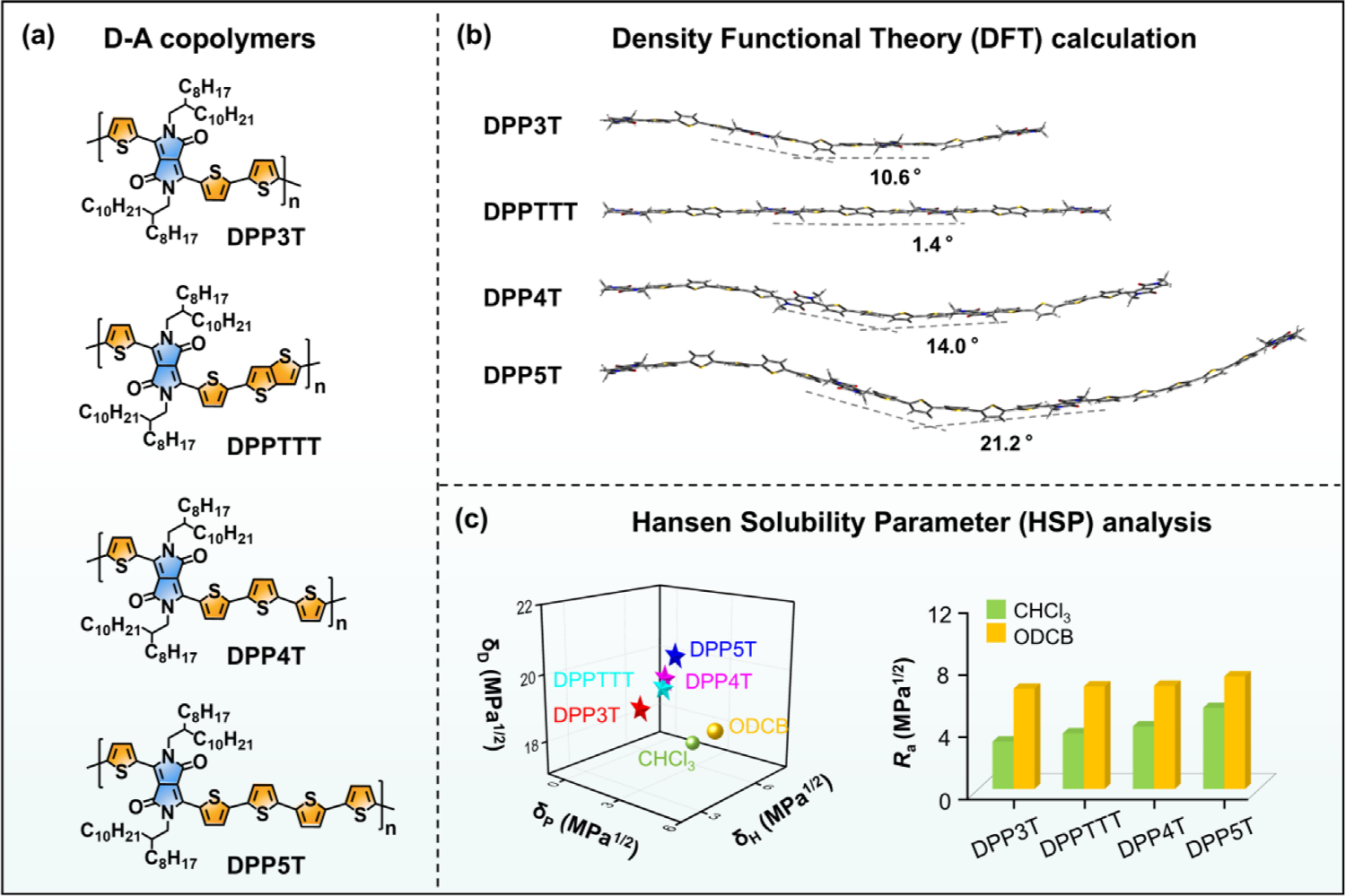
Design strategy of DPP‐based copolymers. (a) Chemical structures of four DPPs (i.e., DPP3T, DPPTTT, DPP4T and DPP5T). (b) DFT‐simulated geometries of the trimers of four DPPs in side view. (c) HSP coordinates of four DPPs and solvents (i.e., CHCl_3_ and ODCB) (left). HSP distance between four DPPs and solvents (i.e., CHCl_3_ and ODCB) (right).

Four DPP‐based copolymers with extended donor units (DPP3T, DPPTTT, DPP4T, and DPP5T) with molecular weights (*M*_n_) of 17.8–29.9 kg/mol (*Ð* = 2.12–3.23) were synthesized via Stille coupling copolymerization [34, 35] (Figures S1–S3 and Table S1), and their molecular structures were illustrated in Figure 1a. To investigate the chain conformation of four DPPs, density functional theory (DFT) calculations based on trimers were performed using the ωB97xD/6‐31G(d,p) basis set [36]. The branch alkyl side chains on the DPP units were simplified to methyl groups to reduce computational time. By calculation, the dihedral angles between the planes of adjacent DPP units were 10.6°, 1.4°, 14.0°, and 21.2° for DPP3T, DPPTTT, DPP4T, and DPP5T, respectively (Figure 1b). Thus, the coplanarity order follows DPPTTT > DPP3T > DPP4T > DPP5T.

In the case of 1st processing strategy, thin films of four DPPs were drop‐cast from two solvents (CHCl_3_ and ODCB), and then investigated by 2D‐GIWAXS schematically described in Figure S4. These two solvents were chosen because of their different selectivity for DPPs, that is, CHCl_3_ is a good solvent for these DPPs, while ODCB is a relatively poor solvent for them. According to the Hansen Solubility Parameter (HSP) analysis, the compatibility between a solvent and a polymer can be quantified by the HSP distance (*R*_a_), in which a larger *R*_a_ means a poorer compatibility between them [37, 38]. By calculation, the *R*_a_ values of DPPs in CHCl_3_ (3.05–5.22 MPa^1/2^) are smaller than those in ODCB (6.45–7.24 MPa^1/2^), indicating that these DPPs had a better compatibility with CHCl_3_ than with ODCB (Figure 1c, see detailed calculation in the Supporting Information). Comparing four DPPs in the same solvent, their *R*_a_ values increased with extended donor units (Figure 1c). This meant that the compatibility between DPPs and solvents decreased with extended donor units, in which DPP3T had the best compatibility with both CHCl_3_ and ODCB, whereas DPP5T exhibited the poorest compatibility with these two solvents.

**FIGURE 2:**
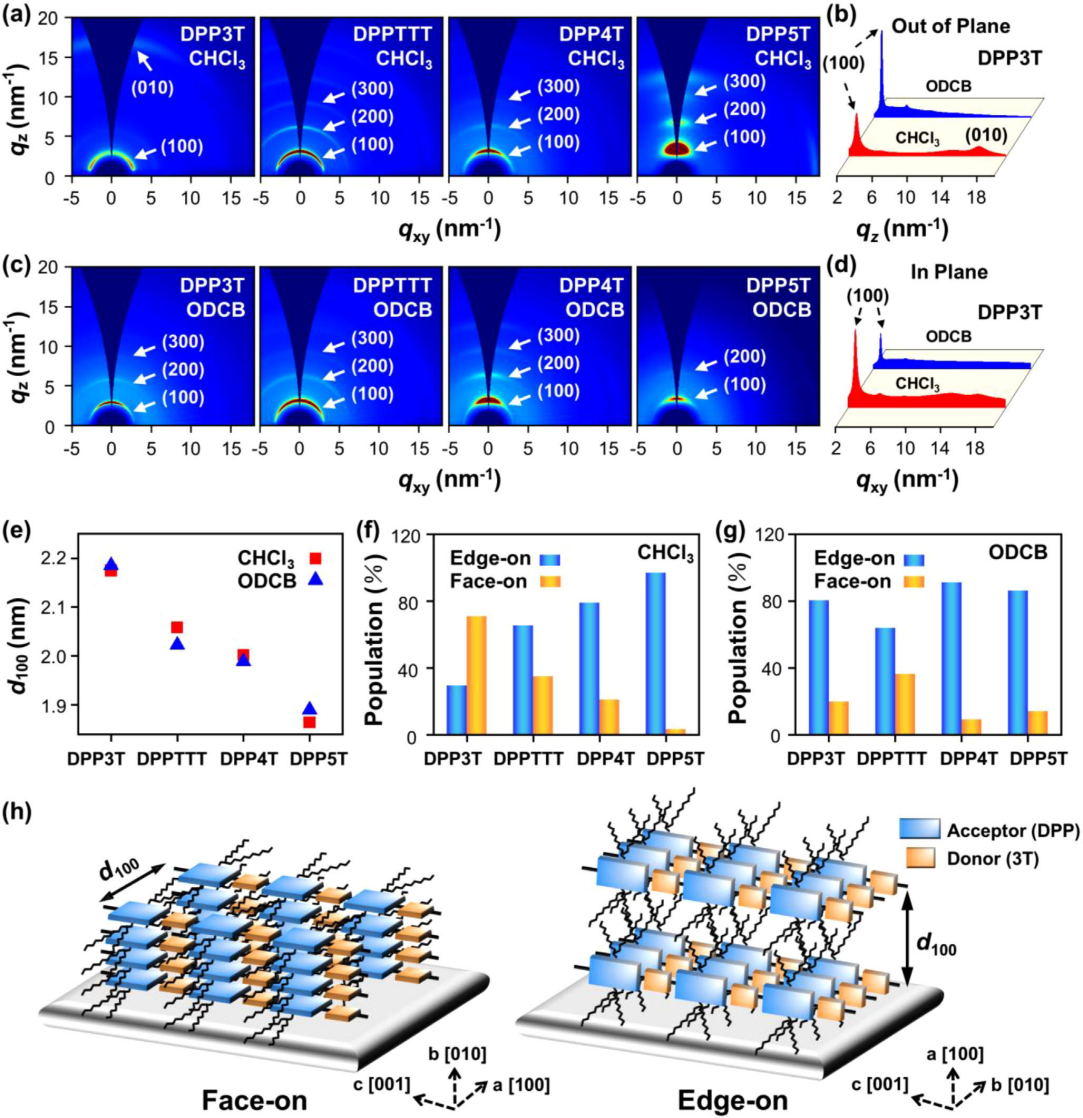
Solid‐state orientation of fabricated polymer films. (a, c) 2D‐GIWAXS images of the four DPP thin films produced from (a) CHCl_3_ and (c) ODCB. (b, d) 1D‐GIWAXS profiles of DPP3T films obtained from the corresponding images along the (b) *q*_z_ and (d) *q*_xy_ directions. *q*_z_: out‐of‐plane direction; *q*_xy_: in‐plane direction. (e) Lamellar spacing along the (100) direction of four DPPs produced from CHCl_3_ and ODCB. (f, g) Edge‐on and face‐on crystal contents in four DPPs produced from (f) CHCl_3_ and (g) ODCB. (h) Schematic of the face‐on and edge‐on orientations in DPPs represented by DPP3T.

When drop‐casted from CHCl_3_, the thin film of DPP3T exhibited a (100) diffraction peak in the in‐plane direction (*q*_xy_) at 2.89 nm^−1^, corresponding to a lamellar spacing (*d*_100_) of 2.17 nm (Figure 2a,e and Table 1). The (010) diffraction peak of DPP3T was clearly observed in the out‐of‐plane direction (*q*_z_ = 16.8 nm^−1^), corresponding to the π–π stacking distance (*d*_010_) of 0.37 nm (Figure 2a,b). This result indicated that DPP3T crystallized in a face‐on orientation, with the alkyl packing and π–π stacking directions being parallel and vertical to the substrate, respectively (Figure 2h, left panel). In contrast, thin films of DPPTTT, DPP4T, and DPP5T drop‐cast from CHCl_3_ displayed a series of (*h*00) diffraction peaks in the *q*_z_ direction (Figure 2a and Figure S5). It proved an edge‐on orientation, where the alkyl packing direction is perpendicular to the substrate and the π–π stacking is parallel to the substrate, respectively (Figure 2h, right panel). The *d*_100_ values of DPPTTT, DPP4T, and DPP5T were 2.06, 2.00, and 1.86 nm, respectively (Figure 2e and Table 1). With an increase in the donor length from 3T to 5T, *d*_100_ decreased slightly. The extended donors of DPPs allowed alkyl side chains of the same volume to achieve sufficient packing with less extension along the lamellar packing (100) direction, resulting in reduced *d*_100_. The full width at half maximum of (100) peaks (FWHM_100_) provide information about the disorder of crystals, and a smaller FWHM_100_ implies a more ordered crystal [39, 40]. DPPTTT had the smallest FWHM_100_ (0.21 nm^−1^) among the four DPPs (0.21–0.57 nm^−1^), implying that it had the most ordered crystalline structure (Table 1). The face‐on and edge‐on crystal contents in various DPPs were calculated by integrating the (100) diffraction peak intensity with χ [41], which is the angle between the substrate normal and the crystal orientation (Figure S6). As per the Ewald sphere correction, the intensity of the integrated (100) peak was multiplied by sin(χ), with χ = 2°−45° and 45°−80° for the edge‐on and face‐on crystals, respectively. DPP3T exhibited a major face‐on orientation of 70.7%, whereas the other three DPPs showed major edge‐on orientations of 65.3%–96.8% (Figure 2f, Table 1, and Figure S7). Clearly, incorporating a longer donor unit into the DPPs favored the formation of the edge‐on over the face‐on in DPP films.

**TABLE 1:** Summary of 2D‐GIWAXS measurements of the DPP films.

					% contributions of χ ^d^	
Sample	Solvent	*d*_100_ (nm) ^a^	FWHM_100_ (nm^−1^) ^b^	*d*_010_ (nm) ^c^	edge‐on (χ = 2°–45°)	face‐on (χ = 45°–80°)
DPP3T	CHCl_3_	2.17	0.42	0.37	29.3	70.7
	ODCB	2.19	0.20	0.38	80.2	19.8
DPPTTT	CHCl_3_	2.06	0.21	0.38	65.3	34.7
	ODCB	2.02	0.27	0.38	63.7	36.3
DPP4T	CHCl_3_	2.00	0.31	0.37	79.0	21.0
	ODCB	1.99	0.29	0.38	91.0	9.0
DPP5T	CHCl_3_	1.86	0.57	0.36	96.8	3.2
	ODCB	1.89	0.40	0.36	86.2	13.8

^a^ Lamellar distance in the (100) direction.

^b^ Full width at half‐maximum (FWHM) of the (100) diffraction peaks.

^c^ π–π stacking distance in the (010) direction.

^d^ Fraction of edge‐on and face‐on crystals in different χ ranges (χ: the angle between the crystal orientation and surface normal). The peak area is divided into χ = 2°–45° and 45°–80° regions that correspond to the edge‐on and face‐on crystals, respectively.

Thin films of these polymers produced from ODCB exhibited some differences compared with those from CHCl_3_. All four DPPs exhibited an edge‐on orientation, as indicated by their (*h*00) diffraction peaks appearing in the *q*_z_ direction (Figure 2c). Their *d*_100_ values are in the range of 1.89–2.19 nm, and the edge‐on crystal contents were in the range of 63.7%–91.0% (Figure 2e, Table 1, and Figure S7). DPPTTT, DPP4T, and DPP5T showed the same crystal orientation when produced from both CHCl_3_ and ODCB solvents. In contrast, the crystal orientation of DPP3T produced from ODCB (edge‐on orientation) was very different from that produced from CHCl_3_ (face‐on orientation). The FWHM_100_ of these DPPs was slightly narrower when drop‐casted from ODCB (0.20–0.40 nm^−1^) than those from CHCl_3_ (0.21–0.57 nm^−1^), implying the formation of more ordered crystalline structures after slower ODCB evaporation (Table 1).

**FIGURE 3:**
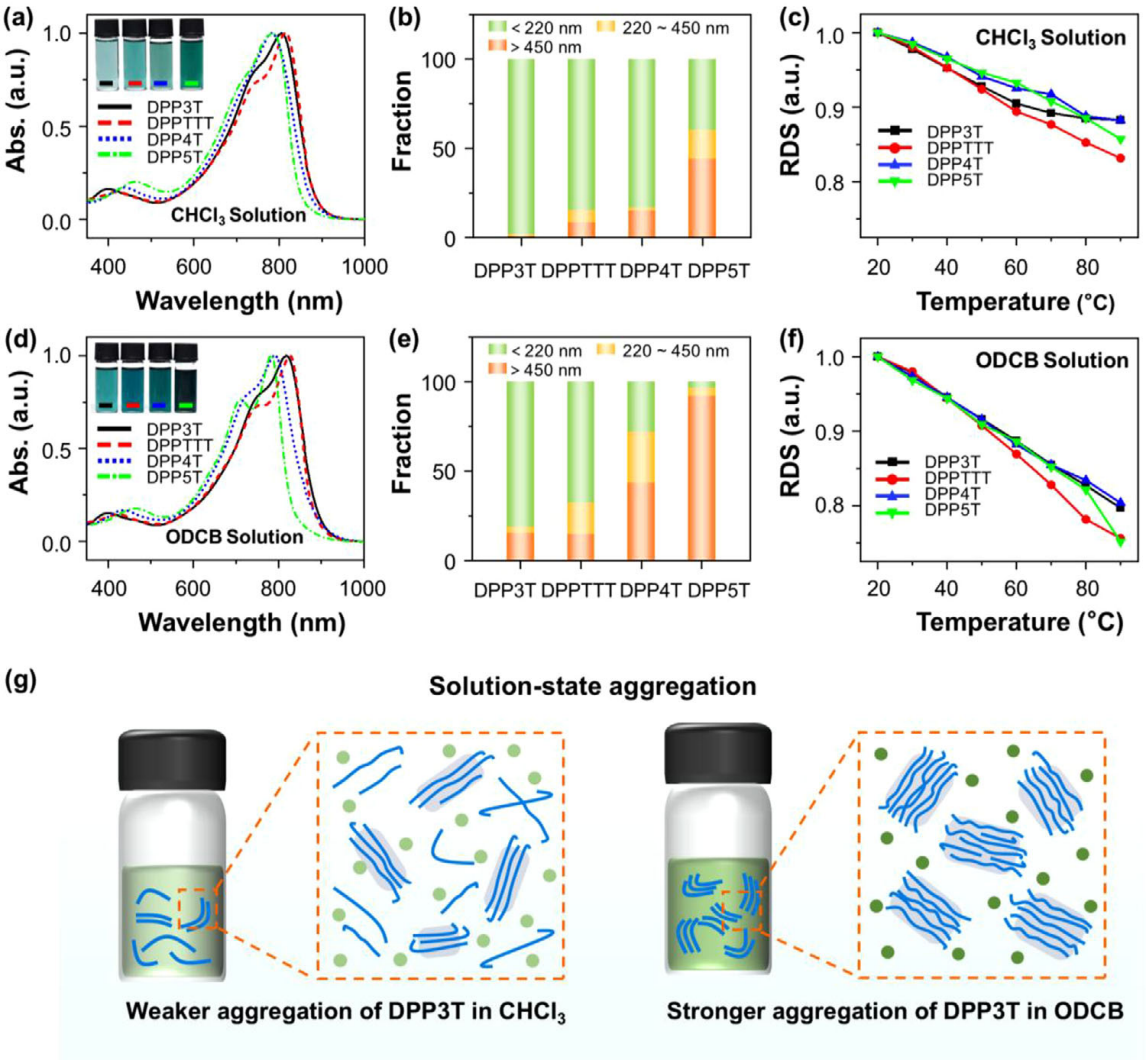
Solution‐state aggregation of DPP‐based copolymers. (a, d) UV–vis absorption spectra of the four DPPs in (a) CHCl_3_ and (d) ODCB solutions. (b, e) Size distribution of four DPPs in (b) CHCl_3_ and (e) ODCB solutions. (c, f) Plots of “relative disaggregation strength” (RDS) of four DPPs in (c) CHCl_3_ and (f) ODCB solutions. The insets in (a) and (d) show the corresponding optical photographs of four DPPs. (g) Schematic illustration of the aggregation of DPPs (represented by DPP3T) in CHCl_3_ and ODCB.

After thoroughly analyzing the crystalline orientation of four DPPs in the film, it is vital to trace back to their solution state and unravel how their solution‐state aggregates, reflected by UV–vis spectroscopy, impacted their crystallite orientation in the film. Four DPPs exhibited a gradual color change from light to deep cyan with an increasing donor length in both CHCl_3_ and ODCB solutions (insets in Figure 3a,d). The color changes were related to the various solution‐state aggregates. In both CHCl_3_ and ODCB, the four DPPs showed two absorption bands at 690–840 nm (band I, ascribed to the intramolecular charge transfer band) and 390–470 nm (band II, ascribed to the π–π* transition absorption band). In CHCl_3_, the 0–0 absorption peaks of DPP3T and DPPTTT obviously red‐shifted to 805 and 814 nm, respectively, compared with those of DPP4T (782 nm) and DPP5T (778 nm), owing to the more planar backbone of DPP3T and DPPTTT (Figure 3a and Table S2), as confirmed by DFT calculation (Figure 1b). The red‐shifted 0–0 absorption peaks of DPP3T and DPPTTT agreed well with the calculated UV absorption using the PBE0/6‐31G(d,p) basis set (Figure S8).

Compared with CHCl_3_ solutions, the 0–0 peaks (782–826 nm) of the DPPs showed obvious red‐shifted (4–13 nm) in the ODCB solution, implying the enhanced coplanarity of DPPs in the latter (Figure 3d). The red‐shift from CHCl_3_ to ODCB became smaller with the extended donor units, in which DPP3T and DPP5T had the largest (13 nm) and smallest (4 nm) red‐shift, respectively (Table S2). It implied that the difference in the solution aggregation between CHCl_3_ and ODCB was larger in DPP3T than DPP5T. At the same time, the 0–1 peak of DPPs red‐shifted from CHCl_3_ (710–751 nm) to ODCB (711–756 nm). The aggregation degree of different DPPs in solutions can be estimated by the intensity ratio of A_0−0_/A_0−1_ absorption peaks [42]. DPP3T had the smallest A_0−0_/A_0−1_ ratio (1.22) in CHCl_3_ among the four DPPs in CHCl_3_ (1.22–1.36) and ODCB (1.26–1.37) solutions, suggesting the weakest aggregation among all eight solutions (Figure 3a,d and Table S2). Notably, in order not to exceed the concentration limit of UV–vis measurements, these DPP solutions were prepared at a lower concentration (0.2 mg/mL), at which the crystallinity of DPPs films produced from the lower concentration decreased compared to those prepared from the higher solution concentration (5 mg/mL), but their crystal orientation remained unchanged (Figure S9).

**FIGURE 4:**
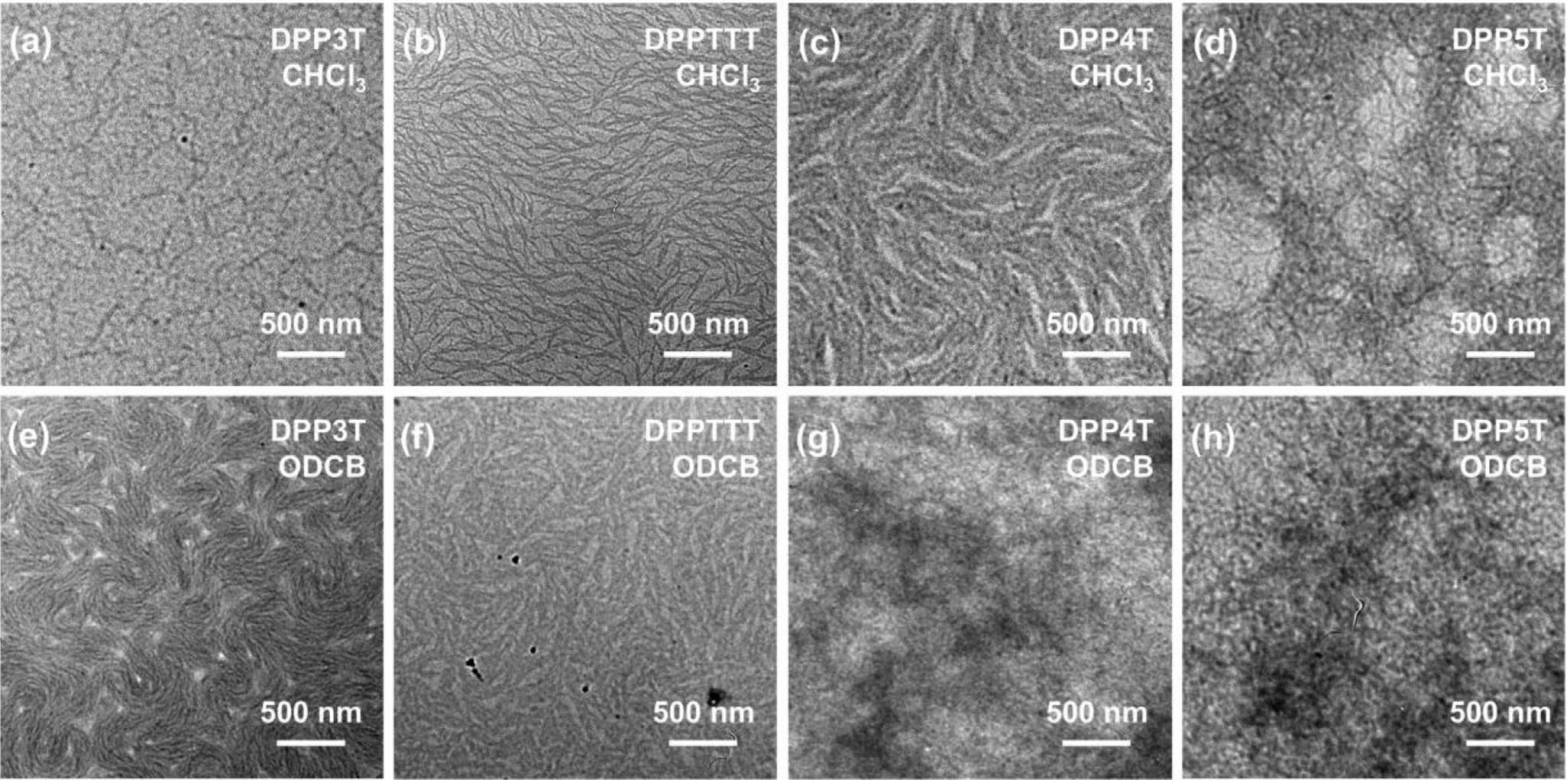
TEM images of four DPP thin films produced from (a–d) CHCl_3_ and (e–h) ODCB.

TEM measurements further corroborated the UV–vis studies. In CHCl_3_, it was evident that the DPPs gradually aggregated with extended donors, transitioning from sparse fibrous morphology for DPP3T to dense fibrous morphology for DPPTTT and DPP4T, and at last an agglomerated morphology for DPP5T (Figure 4a–d). Their aggregation became more pronounced in ODCB (Figure 4e,f). These results further supported that DPP3T aggregated the least among the four DPPs, and they had a stronger aggregation in ODCB than in CHCl_3_. By AFM measurement, the root‐mean‐square (RMS) roughness of the four DPP films cast from CHCl_3_ (0.85–1.60 nm) was smaller than those from ODCB (1.19–2.89 nm) (Figure S10).

To quantify the size of aggregates in different solutions, filtration experiments were performed using filters with the pore sizes of 450 and 220 nm. In CHCl_3_ solutions, DPP3T, DPPTTT, DPP4T, and DPP5T had aggregates (<220 nm) of 98.2, 84.6, 83.0, and 39.8%, respectively (Figure 3b, Figure S11 and Table S3). The aggregates became larger and larger with the increased donor length, among which DPP5T had the highest aggregates (>450 nm) of 44.0%. Moreover, all four DPPs had higher proportion (14.7%–92.0%) of larger aggregates (>450 nm) in ODCB than those in CHCl_3_ (Figure 3e, Figure S12 and Table S3). Such a trend agreed well with the HSP calculations, that is, DPPs had a better compatibility with CHCl_3_ and thus formed smaller aggregates in CHCl_3_ than in ODCB, and DPPs had decreased compatibility with extended donor units and thus DPP5T had the highest fraction of aggregates (>450 nm) in both CHCl_3_ and ODCB. The disaggregation rate of DPPs in CHCl_3_ and ODCB was measured by temperature‐dependent UV−vis spectra. The intensity of the 0−0 peak at increased temperature compared to that of the 0−0 peak at 20 °C was defined as the “relative disaggregation strength (RDS)” (Figure 3c,f). The disaggregation rate could be reflected by the slope of the curves, and a larger slope indicated a higher disaggregation rate [43, 44]. Comparing the disaggregation of the four DPPs in CHCl_3_ and ODCB, all of them showed faster disaggregation in ODCB than those in CHCl_3_, despite their larger aggregates in ODCB. This suggested that the DPPs aggregates in ODCB were more readily broken upon heating (Figure 3c,f and Figure S13).

These results confirm that both the molecular structure (internal factor) of DPPs and solvent (external factor) impacted the aggregation behavior of polymers in the solution, which in turn strongly affected the formation of their crystallite orientation in the solid state. From the perspective of the molecular structure, DPPs with extended donor units exhibited a reduced density of solubilizing alkyl side chains, further decreasing their solubility and thereby leading to progressively intensified aggregation. This was evidenced by the increased *R*_a_ values of four DPPs with extended donor units in both solutions (Figure 1c), more obvious aggregates by TEM (Figure 4), and the increased percentage of aggregates (> 220 nm) in both CHCl_3_ and ODCB solutions in the order of DPP5T > DPP4T > DPPTTT > DPP3T (Figure 3b,e). From the perspective of solvent, four DPPs exhibited stronger aggregation ability in ODCB than in CHCl_3_, as evidenced by the larger *R*_a_ values (6.45–7.24 MPa^1/2^) in ODCB than those in CHCl_3_ (3.05–5.22 MPa^1/2^) (Figure 1c), more pronounced aggregates (Figure 4) and higher percentage of aggregates (>450 nm) in ODCB (Figure 3e).

When these DPPs solutions were drop‐cast to form the films, the crystal orientation in DPP films was influenced by polymer‐polymer (within the aggregates) and polymer‐substrate (between the aggregates and substrate) interactions [14, 29, 45]. In the combination of both molecular structure and solvent effects, DPP3T aggregated the least in CHCl_3_ solution (Figure 3g, left panel). When these DPP3T aggregates interacted with the substrate, the polymer‐substrate interactions dominated over the polymer‐polymer interactions, resulting in an energetically favorable face‐on orientation (Figure 2h, left panel). In contrast, the aggregation of DPP3T was enhanced in ODCB solution (Figure 3g, right panel), and thus preferred to pack into an energetically advantageous edge‐on orientation in the film (Figure 2h, right panel). As for the other three DPPs (i.e., DPPTTT, DPP4T, and DPP5T), they all exhibited a high degree of aggregation in both CHCl_3_ and ODCB, and thereby formed the edge‐on orientation after drop‐casting from both solutions (Figure 2h, right panel).

**FIGURE 5:**
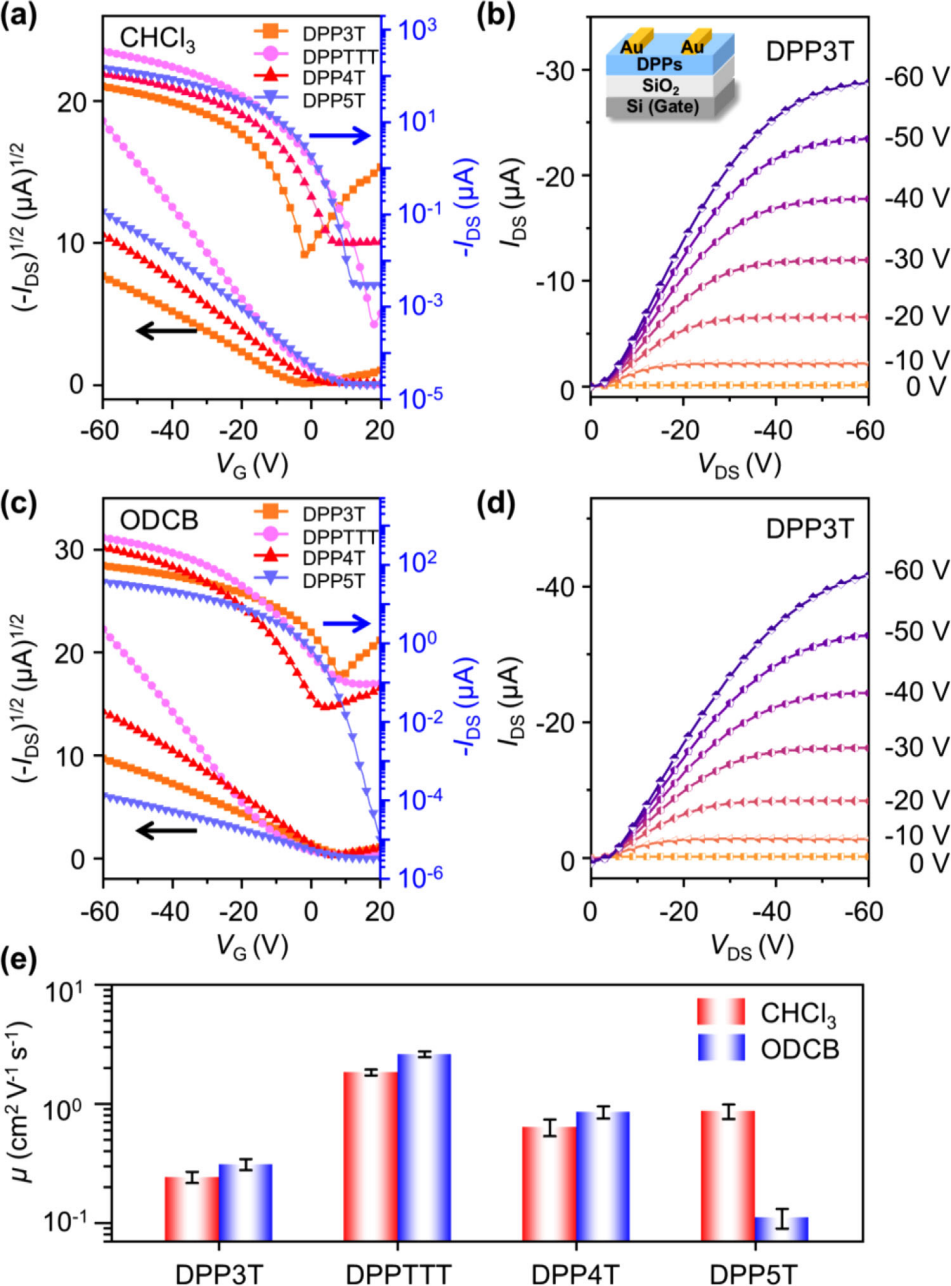
Charge transport properties of fabricated polymer films. (a, c) Transfer curves of the OFETs fabricated by using the four DPPs produced from (a) CHCl_3_ and (c) ODCB. (b, d) Output curves of DPP3T produced from (b) CHCl_3_ and (d) ODCB. The inset in (b) shows the OFET structure. (e) Charge mobilities of the four DPPs produced from CHCl_3_ and ODCB.

After thoroughly understanding the effects of molecular structure and solvents on the solution‐state aggregation and solid‐state crystalline orientation of four DPPs, the relationship between these crystalline structures and their carrier mobilities was built up based on their films as the active layers of OFET devices. DPP3T, DPPTTT, DPP4T, and DPP5T produced from CHCl_3_ showed an average charge mobility (*µ*) of 0.24, 1.84, 0.64, and 0.87 cm^2^ V^−1^ s^−1^, respectively (Figure 5 and Table S4). The highest *µ* of 2.01 cm^2^ V^−1^ s^−1^ was found in DPPTTT. Compared with the films produced from CHCl_3_, DPP3T, DPPTTT, and DPP4T produced from ODCB displayed a slightly increased *µ*, with the average *µ* being 0.31, 2.61, and 0.85 cm^2^ V^−1^ s^−1^, respectively (Figure 5 and Table S4). In contrast, DPP5T produced from ODCB showed a considerably decreased *µ* of 0.11 cm^2^ V^−1^ s^−1^.

The carrier mobilities of the various DPPs were collectively influenced by the molecular structures and crystalline structures. When produced from CHCl_3_, the thin film of DPP3T displayed weaker aggregation in the solution and thus a face‐on orientation in the film (Figure 2h, left panel), in which the π–π stacking direction was normal to the conducting channel, and the in‐plane insulating alkyl side chains further impeded the in‐plane charge transport, leading to degraded device performance. In contrast, the other three DPPs displayed stronger aggregation in the solution and then the edge‐on orientation in the film, which favored carrier transport with π–π stacking parallel to the channel (Figure 2h, right panel). As a result, DPP3T showed a much lower *µ* than the other three DPPs. Among DPPTTT, DPP4T, and DPP5T, DPPTTT had the most ordered crystalline structure, as evidenced by its smallest FWHM_100_ (Table 1). Moreover, DFT calculations and UV–vis absorption features revealed that DPPTTT exhibits a more planar backbone than the other DPPs (Figures 1b, 3a,d). Both effects reduced the energy barrier for the interchain hopping of charge carriers and favored carrier transport in DPPTTT, resulting in the highest *µ*.

In terms of ODCB‐cast thin films, DPP3T, DPPTTT, and DPP4T had stronger aggregation ability in the solution, which was assumed to facilitate charge transport, than those produced from CHCl_3_. As a result, they showed a higher *µ* than the films produced from CHCl_3_. As for DPP5T, it had poor solubility in ODCB in spite of strong aggregation compared with in CHCl_3_, yielding a decreased *µ*. These results presented that the molecular structure of DPPs and solvents synergistically influenced the aggregation of DPPs in solutions and the crystalline structures in the solid film, which in turn impacted their device performance.

### 2.2 Reversible Transformation of Crystal Orientation in DPP3T via Thermal and Solvent Vapor Annealing (2nd Processing Strategy)

**FIGURE 6:**
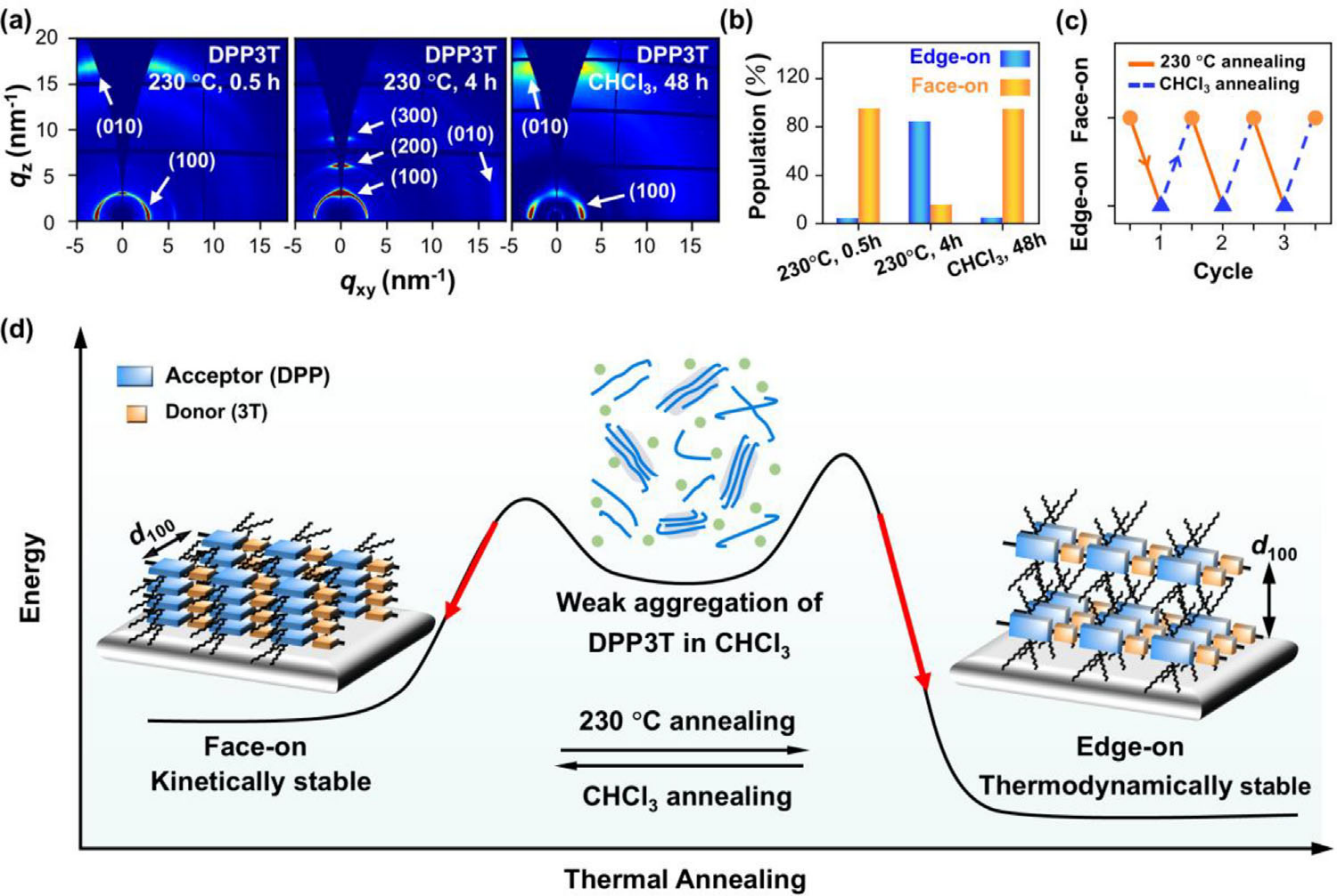
Formation mechanism of different orientation of DPP‐based polymers. (a) 2D‐GIWAXS images of DPP3T thin film produced from CHCl_3_ followed by annealing at 230 °C for 0.5 and 4 h, and then annealed by CHCl_3_ vapor for 48 h in sequence. (b) Edge‐on and face‐on crystal contents in DPP3T under different conditions. (c) Reversible crystal orientation transition in DPP3T after repeated thermal annealing at 230 °C and CHCl_3_ vapor annealing. (d) Schematic of the potential energy of DPP3T aggregation in CHCl_3_ and the crystalline orientation in the solid state under different conditions.

The ability to tailor the transformation between different crystallite orientations in D–A copolymers is important for expanding the effective means of achieving the desired orientation. As DPP3T had different thermodynamically stable states in CHCl_3_ and ODCB (weak and strong aggregation in the solution, corresponding to the face‐on and edge‐on orientations after drop‐casting, respectively), one might naturally consider whether these orientations can be intentionally manipulated through different environmental stimuli. To investigate this, we employed thermal and solvent vapor annealing (2nd processing strategy) to control the crystallite orientation of DPP3T. After the DPP3T thin film produced from CHCl_3_ (exhibiting a face‐on orientation) underwent thermal annealing at 230 °C for 0.5 h, it showed increased crystallinity compared with the drop‐cast state (Figure 6a,b) with the retention of face‐on orientation (95.5%) (Figure S14 and Table S5). After prolonged thermal annealing for 4 h, DPP3T reverted to the edge‐on orientation with the content of 84.5% (Figure 6a,b). The FWHM_100_ decreased from 0.19 nm^−1^ (annealed at 230 °C for 0.5 h, face‐on orientation) to 0.14 nm^−1^ (annealed at 230 °C for 4 h, edge‐on orientation), indicating the more ordered crystalline structure after annealing (Table S5). Interestingly, when the DPP3T film with a newly formed edge‐on orientation was exposed to CHCl_3_ vapor for 48 h, the original face‐on orientation (95.1%) was recovered, with an increased FWHM_100_ of 0.89 nm^−1^ (Figure S14 and Table S5). Unsurprisingly, as the CHCl_3_ molecules required a considerable amount of time to penetrate into the DPP3T film and swell the polymer chains, the change from the edge‐on to face‐on orientation induced by solvent vapor annealing was substantially slower than the reverse transition triggered by thermal annealing. Importantly, such crystallite orientation transition in DPP3T (namely, face‐on to edge‐on based on heating and the reverse transition by subsequent CHCl_3_ vapor annealing) was very reversible and could be switched backward and forward for cycles (Figure 6c and Figure S15).

The driving force to render such reversible transformation is considered to be the mismatch between the thermodynamically stable states of the DPP3T in the CHCl_3_ solution and in the thin film. During thermal and solvent annealing, the thermal‐induced change from face‐on to edge‐on implied that the DPP3T edge‐on crystals were more thermodynamically stable, yet its face‐on crystals were kinetically controlled (Figure 6d). DPP3T aggregated weakly (thermodynamically stable state) in CHCl_3_ solution and formed face‐on orientation (kinetically stable state) in the thin film (Figure 6d, left panel). The edge‐on orientation was not directly formed at this stage because a higher energy barrier needs to be overcome (Figure 6d, right panel). Therefore, the mismatch between their solution and film states rendered the possibility to regulate the crystal orientation in the solid state. The 230 °C annealing drove the DPP3T to overcome the energy barrier to transform into a more stable edge‐on orientation, and the CHCl_3_ vapor annealing assisted the DPP3T change back to kinetically trapped face‐on orientation. For the other three DPPs, their thermodynamically stable state in the solution (i.e., strong aggregation) matched the thermodynamically stable state in the solid state (i.e., edge‐on orientation). Therefore, they retained the edge‐on orientation throughout the thermal annealing process (Figure S16).

By UV–vis measurement, these DPP3T films showed a reversible shift in the position of 0−0 peak and the ratio of A_0−0_/A_0−1_ after alternating thermal annealing (∼825 nm and 0.91, respectively) and CHCl_3_ vapor annealing (∼814 nm and 1.02, respectively) (Figure S17). This suggested the increased effective conjugation length and dominant interchain interaction after thermal annealing, which was consistent with the thermal‐induced increased crystallinity of DPP3T films evidenced by 2D‐GIWAXS data (Figure 6a).

The device performance of the DPP3T thin film during the crystal orientation transition was analyzed (Figure S18 and Table S6). The DPP3T film treated at 230 °C for 0.5 h showed an average *µ* of 0.33 cm^2^ V^−1^ s^−1^. Its *µ* slightly increased owing to increased film crystallinity and ordering of crystalline structures, compared with the *µ* of the film before thermal annealing (0.24 cm^2^ V^−1^ s^−1^). After prolonged thermal treatment for 4 h, DPP3T showed a significantly increased *µ* (0.51 cm^2^ V^−1^ s^−1^). In this situation, DPP3T transformed from face‐on to edge‐on orientation, which is advantageous for charge transport. Simultaneously, the film crystallinity and ordering were further enhanced after thermal annealing. Both changes favored charge transport, yielding the considerably improved *µ*. After CHCl_3_ vapor annealing was performed, DPP3T reverted to the face‐on orientation with the in‐plane insulating alkyl‐side‐chain packing and decreased ordering, as evidenced from a larger FWHM_100_; the *µ* noticeably decreased to 0.23 cm^2^ V^−1^ s^−1^. These results further proved that charge transport in DPP3T films was closely correlated with the crystalline structures.

### 2.3 Synergistic Interplay of Crystal Orientation in DPP‐Based Copolymers via Blending (3rd Processing Strategy)

In addition to the two aforementioned strategies focused on single‐DPP copolymers, blending different DPPs offers a promising route for tailoring the solution‐state aggregation and elucidating the interplay of crystal orientation in DPP‐based copolymers. In what follows, we employed a blending strategy (3rd processing strategy) in which DPP3T and DPP5T (exhibiting weak and strong aggregation in CHCl_3_ solution, and thus face‐on and edge‐on orientations in the film, respectively) were mixed together at different weight ratios to investigate this interplay mechanism.

**FIGURE 7:**
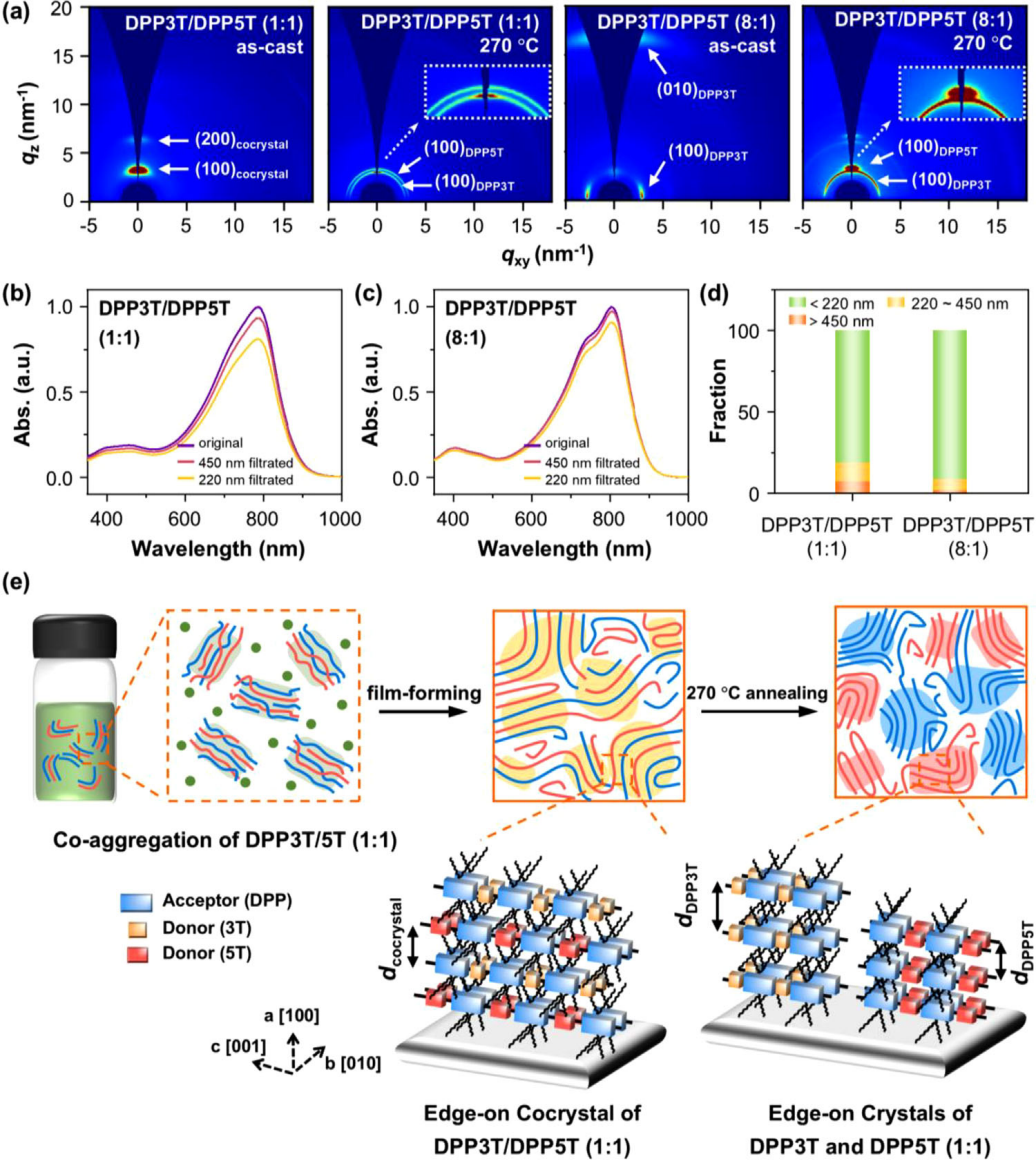
Cocrystallization of different polymers via solution‐state aggregation. (a) 2D‐GIWAXS images of DPP3T/DPP5T blends (weight ratio = 1:1 and 8:1) under different conditions (produced from CHCl_3_ and thermally annealed at 270 °C. (b, c) Absorption spectra in the filtration experiments of DPP3T/DPP5T blends at weight ratio of (b) 1:1 and (c) 8:1 in CHCl_3_. (d) Size distribution of two DPP3T/DPP5T blends in CHCl_3_. (e) Schematic of the formation of the co‐aggregation of DPP3T/DPP5T (1:1) blends in the CHCl_3_ solution and their molecular packing in the solid state. DPP3T and DPP5T co‐aggregate in the CHCl_3_ solution and cocrystallize into edge‐on cocrystals when produced from the solution. After thermal annealing at 270 °C, the cocrystal structures undergo phase separation, forming respective DPP3T and DPP5T crystal domains in the edge‐on orientation.

As shown in Figure 7a, the DPP3T/DPP5T blend at a weight ratio of 1:1 showed only one (100) diffraction peak in the *q*_z_ direction at 3.22 nm^−1^ (*d*_100_ = 1.95 nm) (Table S7), and it was distinct from the pure DPP3T film exhibiting the (100) peak in the *q*_xy_ direction. This indicated that the DPP3T/DPP5T blend formed edge‐on crystals. Moreover, the *d*_100_ of the blend was between those of DPP3T (*d*_100_ = 2.17 nm) and DPP5T (*d*_100_ = 1.86 nm) (Table 1 and Figure S19), suggesting both components affected the crystallization and contributed to the *d*_100_. Because DPP3T and DPP5T have very similar molecular structures, they may form cocrystals with alkyl side chains interdigitating with each other to form a balanced spacing along the *q*_z_ direction (Figure 7e), which can be further corroborated by the following points. The full width at half maximum of (100) peaks (FWHM_100_) of DPP3T/DPP5T blend is 0.39 nm^−1^, which is smaller than both of DPP3T (0.42 nm^−1^) and DPP5T (0.57 nm^−1^) (Table 1 and S7). The reduced FWHM_100_ in the blend indicates a more ordered crystalline packing than respective homopolymers, which helped to exclude the possibility that one polymer crystallized while the other was amorphous. DSC measurements further proved the formation of DPP3T/DPP5T cocrystals. The melting points of DPP3T and DPP5T are 276 °C and 320 °C, respectively (Figure S20). The as‐cast DPP3T/DPP5T (1:1) blend exhibits two adjacent endothermic peaks: a main peak at 300 °C with a shoulder peak at 281 °C (Figure S20). The peak (300 °C) is between the melting points of DPP3T and DPP5T, attributed to the melting point of DPP3T/DPP5T cocrystals. The shoulder peak (281 °C) is attributed to the melting point of DPP3T. During the DSC heating process, because the DPP3T/DPP5T cocrystals were unstable, minor DPP3T chains escaped from the cocrystals and exhibited its own melting point. These experiments revealed that DPP5T had a stronger inducibility than DPP3T, and thus induced the formation of edge‐on cocrystals in the DPP3T/DPP5T (1:1) blend.

By thermal annealing at 270 °C, the DPP3T/DPP5T (1:1) blend film exhibited two (100) diffraction peaks at *q*_z_ = 2.99 (*d*_100_ = 2.10 nm) and 3.31 nm^−1^ (*d*_100_ = 1.90 nm), corresponding to the DPP3T and DPP5T crystal domains, respectively (Figure 7a, Figure S19, and Table S7). It proved on the other way that the as‐cast DPP3T/DPP5T (1:1) blend before 270 °C‐annealing formed cocrystals. Such cocrystals were unstable and phase‐separated by high‐temperature annealing (Figure 7e). When this 270 °C‐annealed DPP3T/DPP5T (1:1) blend was measured by DSC, it showed two completely separated endothermic peaks at 277 °C and 316 °C, corresponding to the melting points of DPP3T and DPP5T, respectively (Figure S20). Because phase separation happened in the DPP3T/DPP5T (1:1) blend after 270 °C annealing, it showed two melting points (277 °C and 316 °C), which were very close to those of their homopolymers (276 °C and 320 °C).

However, the reverse transformation from edge‐on to face‐on in DPP5T induced by DPP3T was not observed, even increasing the fraction of DPP3T in the DPP3T/DPP5T blend to the ratio of 8:1. The DPP3T/DPP5T (8:1) blend showed the crystallization of only DPP3T in the face‐on orientation, as evidenced from the appearance of the (010) peak in the *q*_z_ direction (*q*_z_ = 16.8 nm^−1^, *d*_010_ = 0.37 nm) and the single (100) peak in the *q*_xy_ direction (*q*_xy_ = 2.9 nm^−1^, *d*_100_ = 2.16 nm); the crystallization of the minor DPP5T was not observed owing to its too low amount (Figure 7a and Table S7). After 270 °C annealing, the crystallization of DPP5T was enhanced, evidenced by the appearance of its (100) peak (*q*_z_ = 3.31 nm^−1^, *d*_100_ = 1.90 nm) (Figure 7a and Table S7). Moreover, DPP3T changed from the face‐on to energetically more stable edge‐on orientation, judging from its (100) peak shifted from the *q*_xy_ to *q*_z_ direction. Both DPP3T and DPP5T phase‐separated from each other and crystallized individually.

After demonstrating the transformation of DPP3T from face‐on to edge‐on orientation and formation of DPP3T/DPP5T cocrystals in the blend (1:1), one might naturally think if the influence of DPP5T on DPP3T already existed in the solution or happened during the film‐forming process. UV–vis spectroscopy was performed again to unravel the aggregation state of DPP3T/DPP5T blend (1:1) in the solution. Importantly, the blend (1:1) showed a single 0‐0 absorption peak at 788 nm, which was between that peak of respective DPP3T (805 nm) and DPP5T (778 nm) before blending (Figure 7b). This deviation from their respective 0‐0 peak indicated the aggregation and interaction between DPP3T and DPP5T. Moreover, filtration experiments revealed the DPP3T/DPP5T blend (1:1) had aggregates (>450 nm) of 7.4% (Figure 7d and Table S8), which was much higher than that of single DPP3T in CHCl_3_ (0.8%), but lower than DPP5T (44.0%) (Table S2). Clearly, this proved that the influence of DPP5T on DPP3T existed already in the solution. The co‐aggregation of DPP3T and DPP5T enhanced the aggregation of DPP3T and induced the formation of their cocrystals in the edge‐on orientation (Figure 7e). In contrast, for the DPP3T/DPP5T blend (8:1), the UV–vis spectrum resembled that of the initial state of DPP3T (Figure 7c) with only slightly increased aggregates (>450 nm) of 1.6% compared to DPP3T (Figure 7d and Table S8). It indicated a negligible effect of DPP5T on DPP3T and their co‐aggregation.

The charge mobilities of DPP3T/DPP5T blends under various conditions were analyzed. The DPP3T/DPP5T (1:1) blend with an edge‐on cocrystal structure showed an average *µ* of 0.33 cm^2^ V^−1^ s^−1^ (Figure S21 and Table S8), which was between that of DPP3T (∼0.24 cm^2^ V^−1^ s^−1^) and DPP5T (∼0.87 cm^2^ V^−1^ s^−1^) (Table S4). However, after annealing at 270 °C, the charge mobilities decreased dramatically to 0.07 cm^2^ V^−1^ s^−1^ due to possibly high temperature‐induced aging in the blends and increased grain boundaries after phase separation between DPP3T and DPP5T. Similar trend of thermal‐induced decreased *µ* (from 0.27 to 0.06 cm^2^ V^−1^ s^−1^) was also observed in the DPP3T/DPP5T (8:1) blend (Figure S21 and Table S9).

## 3 Conclusion

In summary, we tuned the solution aggregates and solid‐state crystallite orientations (face‐on and edge‐on), including their formation, transformation, and synergistic interplay in a series of DPP‐based copolymers (DPP3T, DPPTTT, DPP4T, and DPP5T) by applying different processing, and established a strong connection among their solution‐state aggregates, solid‐state crystalline orientations, and charge‐transport properties. After drop‐casting (1st processing strategy), DPP3T formed face‐on and edge‐on orientations in the films when produced from CHCl_3_ and ODCB, respectively, whereas the other three copolymers (DPPTTT, DPP4T, and DPP5T) exhibited edge‐on orientations when produced from both solvents. DPPs with extended donor units (reduced side‐chain density) and the use of poor solvents enhance solution‐state aggregation, thereby driving the crystalline orientation from face‐on to edge‐on. Thermal and solvent vapor annealing (2nd processing strategy) enabled a highly reversible transition between the face‐on and edge‐on orientations in the films of DPP3T, again confirming the important role of solution aggregates for the controlling of the crystal orientation in the solid state.

Interestingly, blending (3rd processing strategy) DPP3T and DPP5T (exhibiting weak and strong aggregation in CHCl_3_, respectively) in a ratio of 1:1 strengthened the aggregation of DPP3T and induced their co‐aggregation in the solution, driving the transformation of DPP3T from the face‐on to edge‐on orientation and formed their edge‐on cocrystals in the film. Critically, the various crystalline structures of DPPs and their charge mobilities showed a strong relationship, revealing the collective impact of the molecular structure, solution‐state aggregates, and crystallite orientation on charge transport. Overall, this study comprehensively demonstrates how the solution aggregates control the solid‐state crystallite orientation of DPPs, and it can be easily extended to other conjugated polymers in interest, thus providing a deeper understanding on the crystalline behavior of D–A copolymers and their structure–property relationships, underpinning their advances for applications in diverse optoelectronic devices.

## 4 Experimental Section

### 4.1 Materials

3,6‐Bis(5‐bromothiophene‐2‐yl)‐2,5‐bis(2‐octyldodecyl)pyrrolo[3,4‐*c*]pyrrole‐1,4(2H,5H)‐dione (DPP2OD‐Br_2_), 2,5‐bis(trimethylstannyl)thiophene, 2,5‐bis(trimethylstannyl)thieno[3,2‐b]thiophene, 5,5'‐bis(trimethylstannyl)‐2,2'‐bithiophene, and 5,5''‐bis(trimethylstannyl)‐2,2':5',2''‐terthiophene were purchased from Nanjing Zhiyan Technology Co., Ltd. Tri‐(*o*‐tolyl)phosphine [P(*o*‐tol)_3_] and tris(dibenzylideneacetone)dipalladium (0) [Pd_2_(dba)_3_] were obtained from Macklin. All reagents were utilized as received.

### 4.2 Synthesis of DPP‐Based Copolymers

Four DPP‐based copolymers, DPP3T, DPPTTT, DPP4T, and DPP5T, with molecular weights (*M*_n_) of 17.8–29.9 kg/mol (*Ð* = 2.12–3.23) were synthesized via Stille coupling copolymerization [34, 35] (Figures S1–S3 and Table S1). The synthesis procedure for DPP3T is provided as an example. A mixture of DPP2OD‐Br_2_ (254.8 mg, 0.25 mmol), 2,5‐bis(trimethylstannyl)thiophene (102.4 mg, 0.25 mmol), P(*o*‐tol)_3_ (6.1 mg, 0.02 mmol), and Pd_2_(dba)_3_ (4.6 mg, 0.005 mmol) was dissolved in chlorobenzene (18 mL) in a microwave glass tube and then stirred for 10 min. The tube was placed in a microwave reactor, heated at 150 °C for 0.5 h, and then end‐capped with bromobenzene (14 mg, 0.088 mmol) at 150 °C for 10 min. Subsequently, the reaction mixture was cooled to 55 °C and then poured into methanol while stirring for 1 h. The products were sequentially purified by performing Soxhlet extraction using methanol, acetone, *n*‐hexane, and CHCl_3_ to remove possible oligomers and impurities. The product was concentrated in CHCl_3_, precipitated in methanol, and dried under vacuum overnight. The other DPPs were synthesized in a similar manner.

### 4.3 Density Functional Theory (DFT) Calculations

Density functional theory (DFT) calculations were performed using the Gaussian 03 program. The optimization of molecular geometries was carried out with the ωB97xD/6‐31G(d,p) basis set [36]. Calculated UV–vis absorption were conducted at the PBE0/6‐31G(d,p) basis set [46].

### 4.4 Characterization

The GPC curves of DPPs were obtained from a high‐temperature system of Agilent 1260 Infinity II, using polystyrene standards and 1,2,4‐trichlorobenzene as the eluent at 160 °C. ^1^H NMR spectra were recorded on a Bruker spectrometer at 500 MHz in C_2_D_2_Cl_4_ at 120 °C. Two‐dimensional grazing‐incidence wide‐angle x‐ray scattering (2D‐GIWAXS) was performed on BL14B1 and BL02U2 beamlines at the Shanghai Synchrotron Radiation Facility (x‐ray wavelength: 1.24 Å; incidence angle: 0.15°). The polymer concentration for 2D‐GIWAXS measurement was 5 mg/mL. X‐ray diffraction (XRD) was performed by using a D8 ADVANCE x‐ray diffractometer (Bruker) with Cu Kα radiation (*λ* = 1.54 Å). Differential scanning calorimetry (DSC) was performed on a TA DSC Q250 in a nitrogen atmosphere. Transmission electron microscopy (TEM) images were recorded via a Hitachi HT7800 microscope operating at 80 kV. Atomic force microscopy (AFM) images were obtained using an Oxford Cypher VRS 1250 in the tapping mode. Ultraviolet–visible (UV–vis) spectroscopy was obtained from a PerkinElmer Lambda 1050+ UV–vis spectrophotometer. The polymer concentration for UV–vis measurement was 0.2 mg/mL. The current–voltage (*I–V*) curves of the OFETs were evaluated by a Keithley 4200–SCS system in a glovebox of Ar atmosphere. The film thickness of the DPPs used in OFETs is ∼300–400 nm.

## Author Contributions

**Hao Zheng**: investigation, methodology, visualization, writing – original draft. **Hao Zhan**: investigation, writing – review and editing. **Xuebing Luo**: investigation, writing – review and editing. **Juan Peng**: investigation, conceptualization, methodology, funding acquisition, supervision, writing – original draft, writing – review and editing, resources, project administration. **Lixin Li**: investigation, writing – original draft, methodology, visualization. **Yanan Guo**: investigation, writing – review and editing. **Jian Pei**: project administration, writing – review and editing, supervision, conceptualization. **Bingjie Wu**: investigation, writing – review and editing. **Ze‐Fan Yao**: investigation, writing – review and editing. **Yongjie Dong**: investigation, writing – review and editing.

## Conflicts of Interest

The authors declare no conflicts of interest.

## Supporting information




**Supporting File**: advs77363‐sup‐0001‐SuppMat.pdf.

## Data Availability

The data that supports the findings of this study are available in the supplementary material of this article.
