# Supplementary material for: Formation, Transformation, and Synergistic Interplay of Crystalline Orientation in Conjugated Polymers via Solution‐State Aggregation Control
Source: Adv Sci (Weinh). 2026 Aug 25;13(56):e77363. doi: 10.1002/advs.77363 (PMC13505466; doi:10.1002/advs.77363)
Supplement: Supplementary file 1 [file ADVS-13-e77363-s001.pdf]

Supporting Information

Formation, Transformation, and Synergistic Interplay of Crystalline Orientation in Conjugated Polymers via Solution-State Aggregation Control

Hao Zheng#,[1] Lixin Li#,[1] Yanan Guo,[1] Bingjie Wu,[1] Xuebing Luo,[1] Hao, Zhan,[1] Yongjie Dong,[1] Ze-Fan Yao,[2] Juan Peng[1]* and Jian Pei[2]*

[1] State Key Laboratory of Molecular Engineering of Polymers, Department of Macromolecular Science, Fudan University, Shanghai 200438, China

[2] Beijing National Laboratory for Molecular Sciences (BNLMS), Key Laboratory of Polymer Chemistry and Physics of Ministry of Education, Center of Soft Matter Science and Engineering, College of Chemistry and Molecular Engineering, Peking University, Beijing 100871, China

# H. Zheng and L. Li contributed equally to this work.

Corresponding Authors

E-mails: juanpeng@fudan.edu.cn (J. Peng); jianpei@pku.edu.cn (J. Pei)

The Supporting Information file includes:

1. Hansen Solubility Parameter (HSP) Calculation

2. OFET Device Fabrication

3. Table S1-S9

4. Figure S1-S21

1. Hansen Solubility Parameter (HSP) Calculation.

To gain insight into the polymer-solvent interaction, the Hansen Solubility Parameter (HSP) analysis was employed. The HSP is composed of the dispersion interaction (δ_D), polar interaction (δ_P), and hydrogen-bonding interaction (δ_H) [1,2]. The HSP distance (R_a) can be used to assess the compatibility between a polymer and a solvent using Equation 1 [2]:

$$R_a = \left[4(\delta_{D1} - \delta_{D2})^2 + (\delta_{P1} - \delta_{P2})^2 + (\delta_{H1} - \delta_{H2})^2\right]^{1/2} \quad (1)$$

A smaller R_a means better compatibility between the polymer and the solvent. The soluble sphere of DPP3T, DPPTTT, DPP4T, and DPP5T is centered at (18.5, 0.4, 5.5) $MPa^{1/2}$, (19.0, 0.6, 6.6) $MPa^{1/2}$, (19.3, 0.6, 6.7) $MPa^{1/2}$, and (19.9, 0.5, 7.4) $MPa^{1/2}$, respectively. The soluble sphere of $CHCl_3$ and ODCB is centered at (17.8, 3.1, 5.7) $MPa^{1/2}$ and (19.2, 6.3, 3.3) $MPa^{1/2}$, respectively. By calculation, the R_a values of DPPs in $CHCl_3$ are 3.05, 3.58, 4.03, and 5.22 $MPa^{1/2}$ for DPP3T, DPPTTT, DPP4T, and DPP5T, respectively. The R_a values of DPPs in ODCB are 6.45, 6.60, 6.64, and 7.24 $MPa^{1/2}$ for DPP3T, DPPTTT, DPP4T, and DPP5T, respectively. Comparing four DPPs in $CHCl_3$ and ODCB, their R_a values in $CHCl_3$ (3.05–5.22 $MPa^{1/2}$) are smaller than those in ODCB (6.45–7.24 $MPa^{1/2}$), suggesting that these DPPs had a better compatibility with $CHCl_3$ than with ODCB. Moreover, comparing four DPPs in the same solvent, their R_a values increased with extended donor units. It meant that the compatibility between DPPs and solvents decreased with extended donor units, in which DPP3T and DPP5T had the best and poorest compatibility in both $CHCl_3$ and ODCB, respectively.

2. OFET Device Fabrication.

Bottom-gate/top-contact OFET devices were fabricated on Si/SiO_2 wafers. The substrates were cleaned by an ultrasonic cleaner with deionized water, acetone and isopropanol, then dried under a nitrogen flow, followed by plasma treatment for 10 min and then modified with n-octadecyltrichlorosilane. The DPPs copolymers were dissolved in $CHCl_3$ or ODCB (5 mg/mL), then spin-coated on pre-cleaned wafers. Au layer (60 nm) was vacuum-evaporated on the silicon substrates as source and drain electrodes with an interdigitated electrode pattern (width = 300 μm, length = 30 μm). The field-effect mobility (μ) was extracted in the saturation regime using the relationship $I_{DS} = W/(2L)C_g\mu(V_G - V_T)^2$, where I_{DS} is the saturation drain current, C_g is the capacitance of SiO_2 dielectric (C_g = 11.5 nF/cm^2), V_G and V_T are the gate and threshold voltages [3]. The device performance was evaluated in argon atmosphere using a Keithley 4200-SCS semiconductor characterization system.

References.

1. D. L. Ho, C. J. Glinka, "New insights into Hansen's Solubility Parameters." *J. Polym. Sci. B* **2004**, *42*, 4337−4343.
2. K. Zhao, Q. Zhang, L. Chen, T. Zhang, Y. Han, "Nucleation and Growth of P(NDI2OD-T2) Nanowires via Side Chain Ordering and Backbone Planarization." *Macromolecules* **2021**, *54*, 2143−2154.
3. H, H. Choi, K. Cho, C. D. Frisbie, H. Sirringhaus, V. Podzoroy, "Critical Assessment of Charge Mobility Extraction in FETs." *Nat. Mater.* **2018**, *17*, 2−7.

3. Table S1-S9

Table S1. Summary of Molecular Weights (M_n) and Dispersity ($Đ$) of DPPs.

Sample	M_n (kg/mol)	$Đ$
DPP3T	20.8	3.23
DPPTTT	24.5	2.78
DPP4T	29.9	2.67
DPP5T	17.8	2.12

Table S2. Summary of UV-vis Spectra of DPPs in the Solution State.

Sample	Solvent	λ_{0-0} (nm)	λ_{0-1} (nm)	$\lambda_{high\ energy}$ (nm)	A_{0-0}/A_{0-1}
DPP3T	$CHCl_3$	805	751	398	1.22
	ODCB	818	755	398	1.26
DPPTTT	$CHCl_3$	814	748	422	1.35
	ODCB	826	756	423	1.37
DPP4T	$CHCl_3$	782	722	438	1.36
	ODCB	791	729	436	1.26
DPP5T	$CHCl_3$	778	710	463	1.35
	ODCB	782	711	463	1.31

Table S3. Summary of the Size of Aggregates from the Filtration Experiments.

Sample	Solvent	Percentages of aggregate size (%)		
		> 450 nm	220 nm ~ 450 nm	< 220 nm
DPP3T	$CHCl_3$	0.8	1.0	98.2
	ODCB	15.5	3.4	81.1
DPPTTT	$CHCl_3$	8.4	7.0	84.6
	ODCB	14.7	17.8	67.5
DPP4T	$CHCl_3$	15.2	1.8	83.0
	ODCB	43.5	28.6	27.9
DPP5T	$CHCl_3$	44.0	16.2	39.8
	ODCB	92.0	4.6	3.4

Table S4. Summary of OFET Device Performances of DPP Films Drop-cast from Different Solvents.

Sample	Solvent	μ_{avg} (cm^2 V^{-1} s^{-1})[a]	μ_{max} (cm^2 V^{-1} s^{-1})[b]	V_{th} (V)	I_{on}/I_{off}
DPP3T	$CHCl_3$	0.24 ± 0.03	0.29	4 ~ 16	$10^2 \sim 10^3$
	ODCB	0.31 ± 0.03	0.35	1 ~ 12	$10^2 \sim 10^3$
DPPTTT	$CHCl_3$	1.84 ± 0.11	2.01	-2 ~ 7	$10^3 \sim 10^4$
	ODCB	2.61 ± 0.14	2.82	-15 ~ 5	$10^3 \sim 10^6$
DPP4T	$CHCl_3$	0.64 ± 0.10	0.74	1 ~ 8	$10^3 \sim 10^4$
	ODCB	0.85 ± 0.10	1.01	1 ~ 9	$10^3 \sim 10^4$
DPP5T	$CHCl_3$	0.87 ± 0.12	1.04	1 ~ 10	$10^3 \sim 10^4$
	ODCB	0.11 ± 0.02	0.13	9 ~ 17	$10^5 \sim 10^6$

[a] Average charge carrier mobility. [b] Maximum charge carrier mobility.

Table S5. Summary of 2D-GIWAXS Measurements of DPP3T Films under Different Conditions.

Sample		d_{100} (nm)[a]	$FWHM_{100}$ (nm^{-1})[b]	% contributions of χ[c]	
				edge-on (χ=2-45°)	face-on (χ=45-80°)
DPP3T	230 °C, 0.5 h	2.10	0.19	4.5	95.5
	230 °C, 4 h	2.05	0.14	84.5	15.5
	$CHCl_3$, 48 h	2.07	0.89	4.9	95.1

[a] Lamellar distance in the (100) direction. [b] Full width at half-maximum (FWHM) of the (100) diffraction peaks. [c] Quantification of *edge-on* and *face-on* crystal contents in different χ ranges (χ: the angle between the crystal orientation and surface normal). The peak area is divided into χ = 2–45° and 45–80° regions that correspond to the edge-on and face-on crystals, respectively.

Table S6. Summary of OFET Device Performances of DPP3T Films under Different Conditions.

Sample		μ_{avg} (cm^2 V^{-1} s^{-1})[a]	μ_{max} (cm^2 V^{-1} s^{-1})[b]	V_{th} (V)	I_{on}/I_{off}
DPP3T	230 °C, 0.5 h	0.33 ± 0.05	0.41	9 ~ 14	$10^4 \sim 10^6$
	230 °C, 4 h	0.51 ± 0.09	0.58	9 ~ 18	$10^3 \sim 10^8$
	$CHCl_3$, 48 h	0.23 ± 0.03	0.25	-25 ~ 1	$10^4 \sim 10^5$

[a] Average charge carrier mobility. [b] Maximum charge carrier mobility.

Table S7. Summary of 2D-GIWAXS Measurements of DPP3T/DPP5T Blended Films under Different Conditions.

Samples		d_{100} (nm)[a]	$FWHM_{100}$ (nm^{-1})[b]
DPP3T/DPP5T (1:1)	as-cast	1.95	0.39
	270 °C	2.10 / 1.90	0.14 / 0.13
DPP3T/DPP5T (8:1)	as-cast	2.16	0.32
	270 °C	2.10 / 1.90	0.13 / 0.15

[a] Lamellar distance in the (100) direction. [b] Full width at half-maximum (FWHM) of the (100) diffraction peaks.

Table S8. Summary of the Size of Aggregates in DPP3T/DPP5T Blends from the Filtration Experiments.

Sample	Solvent	Percentages of aggregate size (%)		
		> 450 nm	220 nm ~ 450 nm	< 220 nm
DPP3T/DPP5T (1:1)	$CHCl_3$	7.4	11.5	81.1
DPP3T/DPP5T (8:1)	$CHCl_3$	1.6	7.0	91.4

Table S9. Summary of OFET Device Performances of DPP3T/DPP5T Blended Films under Different Conditions.

Sample		μ_{avg} ($cm^2 V^{-1} s^{-1}$)[a]	μ_{max} ($cm^2 V^{-1} s^{-1}$)[b]	V_{th} (V)	I_{on}/I_{off}
DPP3T/DPP5T (1:1)	as-cast	0.33 ± 0.09	0.46	-5 ~ 1	$10^2 \sim 10^5$
	270 °C	0.07 ± 0.02	0.12	-3 ~ 0	$10^5 \sim 10^6$
DPP3T/DPP5T (8:1)	as-cast	0.13 ± 0.08	0.27	3 ~ 8	$10^5 \sim 10^6$
	270 °C	0.06 ± 0.02	0.11	-27 ~ 6	$10^4 \sim 10^6$

[a] Average charge carrier mobility. [b] Maximum charge carrier mobility.

4. Figure S1-S21

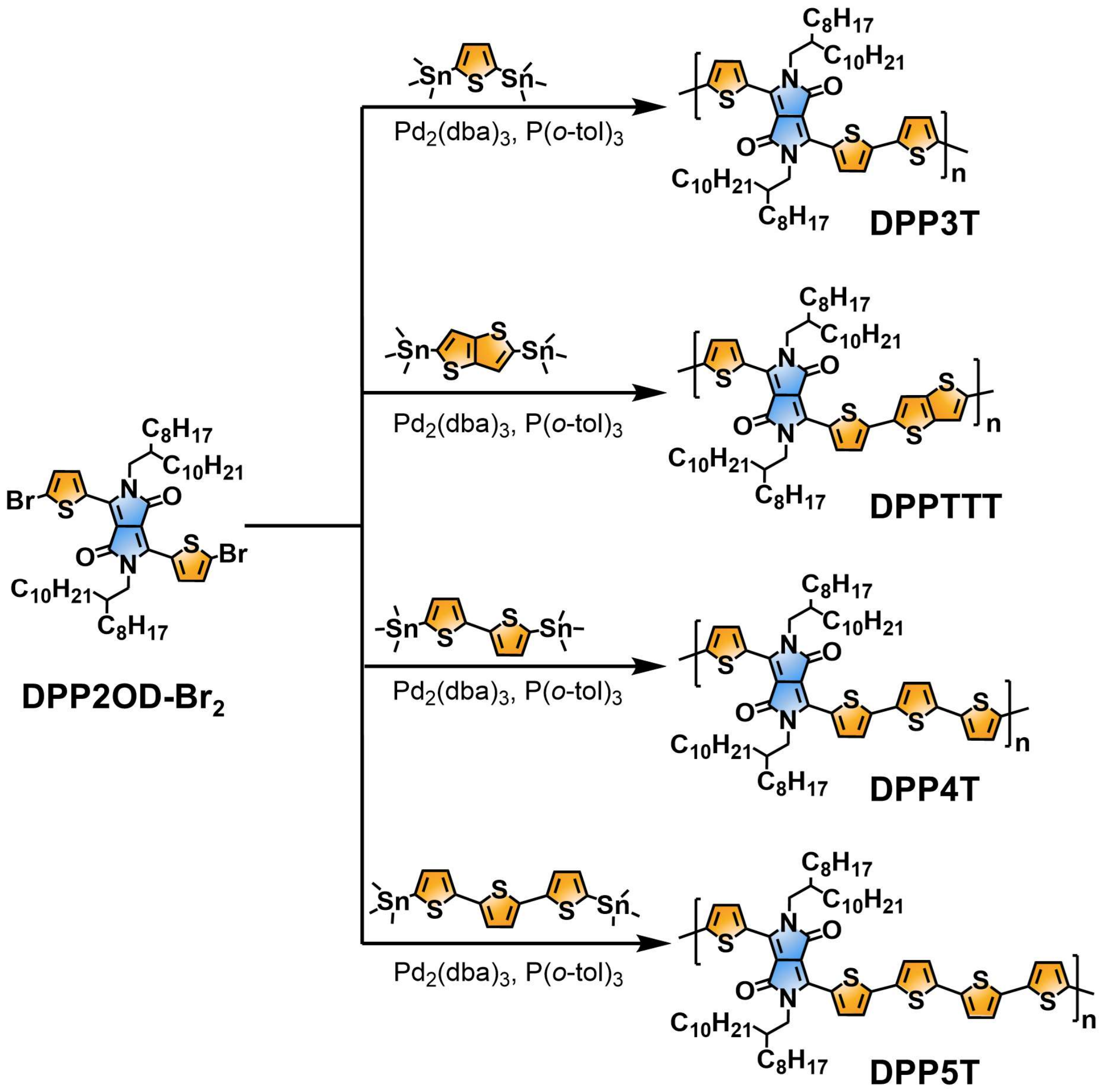

Figure S1. Synthesis route for four DPPs carrying different donor units via Stille coupling copolymerization.

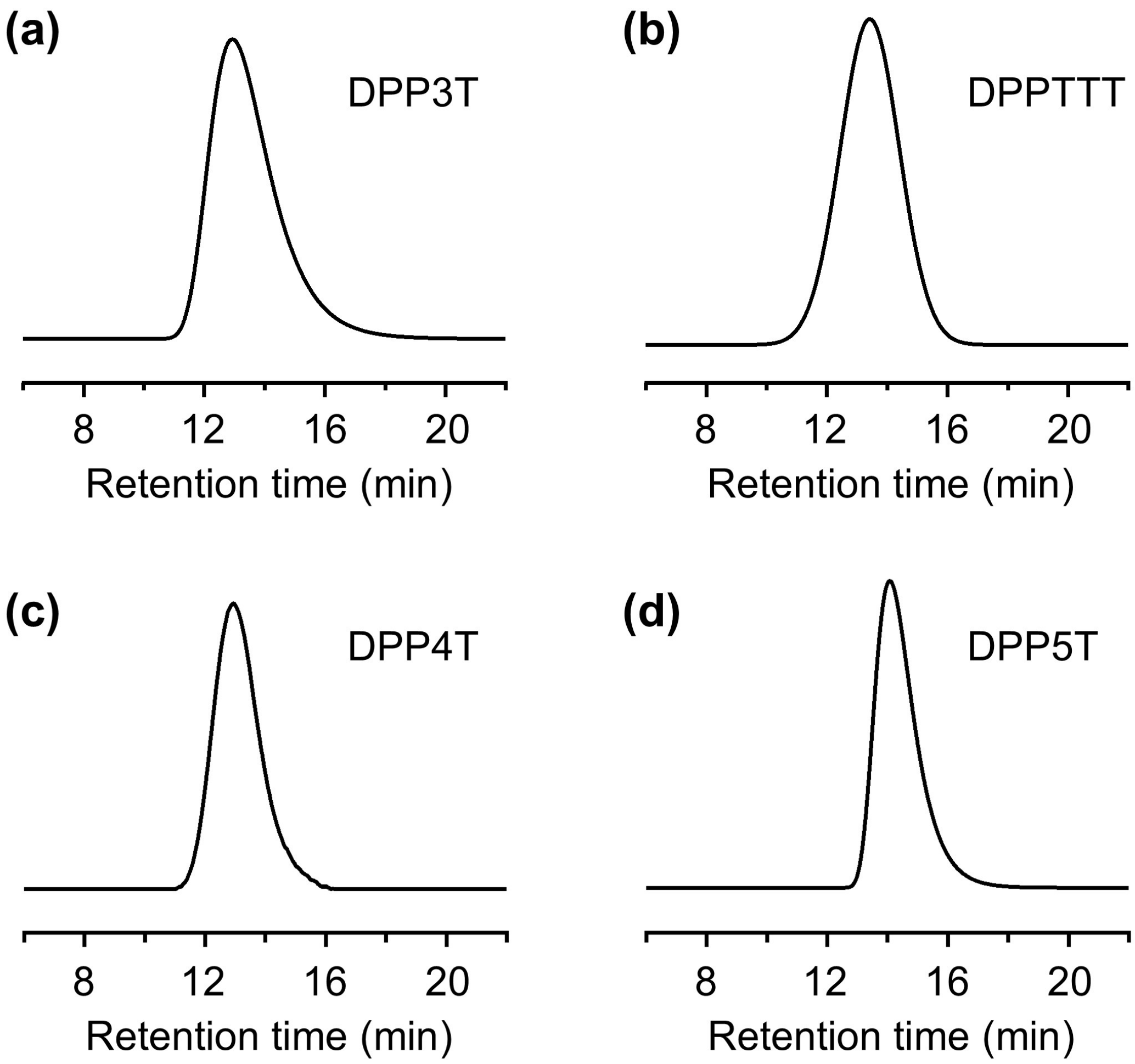

Figure S2. GPC profiles of (a) DPP3T, (b) DPPTTT, (c) DPP4T, and (d) DPP5T, respectively.

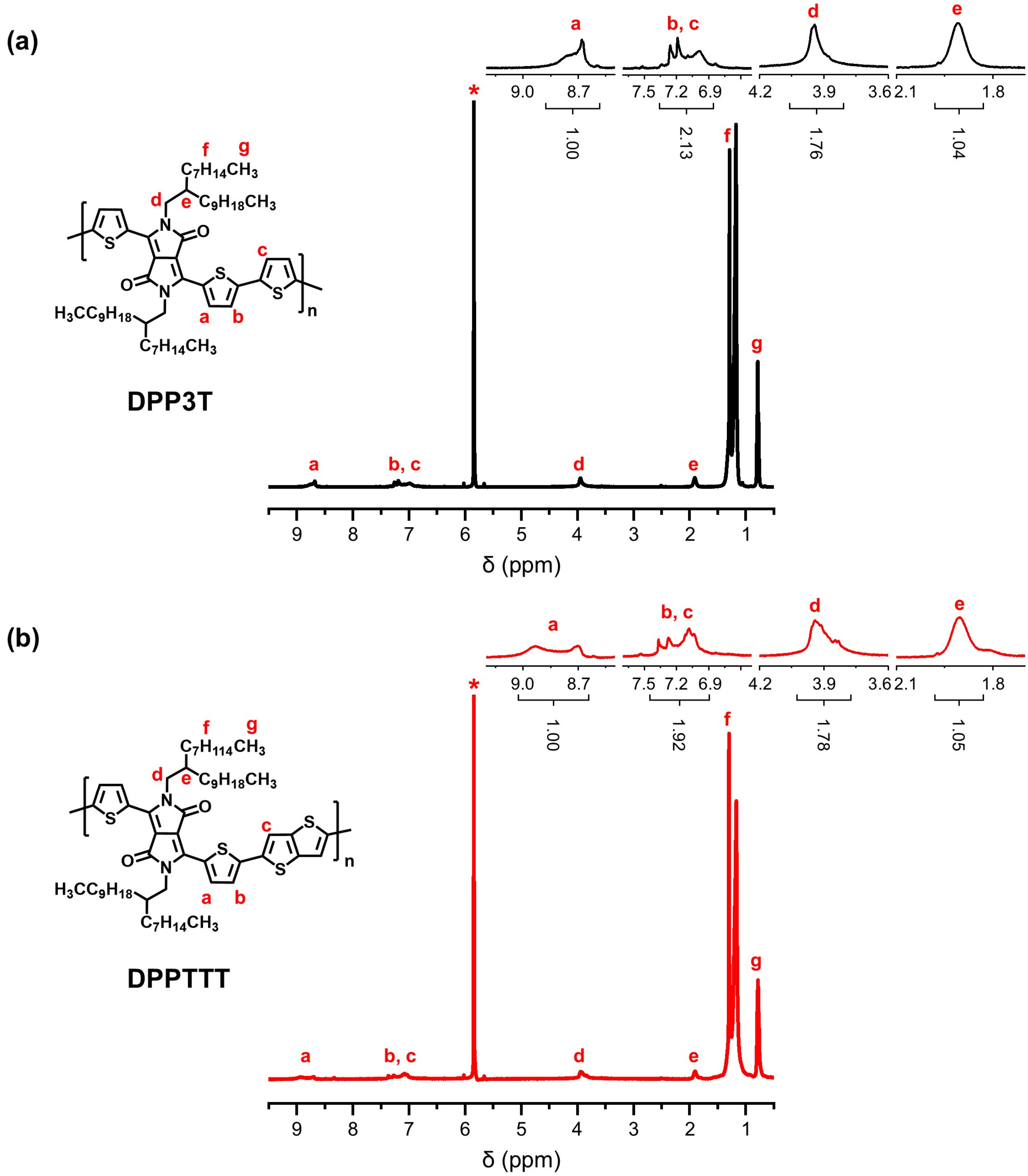
(a)
DPP3T
a
b, c
d
e
f
g
*
9.0
8.7
7.5
7.2
6.9
4.2
3.9
3.6
2.1
1.8
1.00
2.13
1.76
1.04
9
8
7
6
5
4
3
2
1
δ (ppm)
(b)
DPPTTT
1.00
1.92
1.78
1.05

(c)

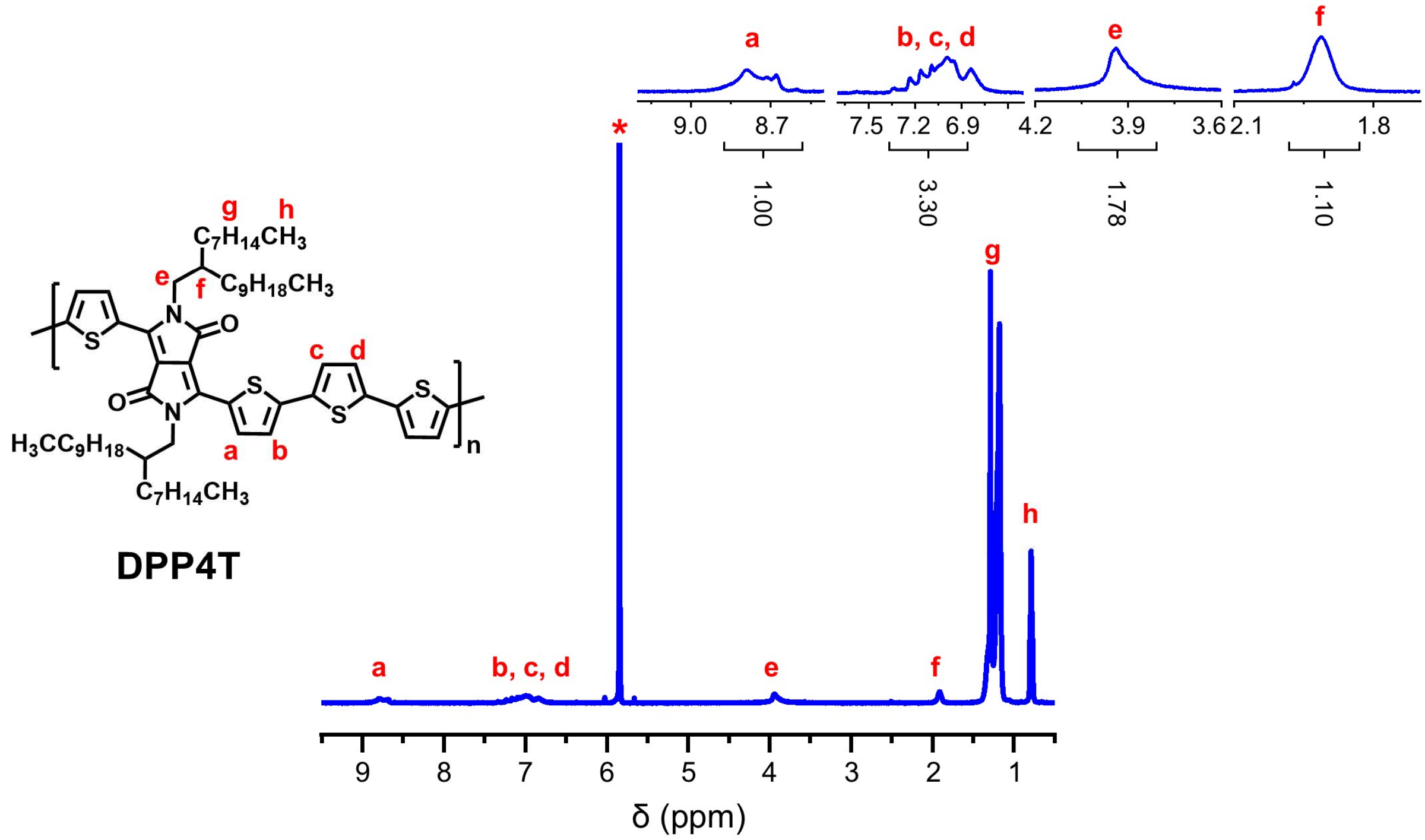

(d)

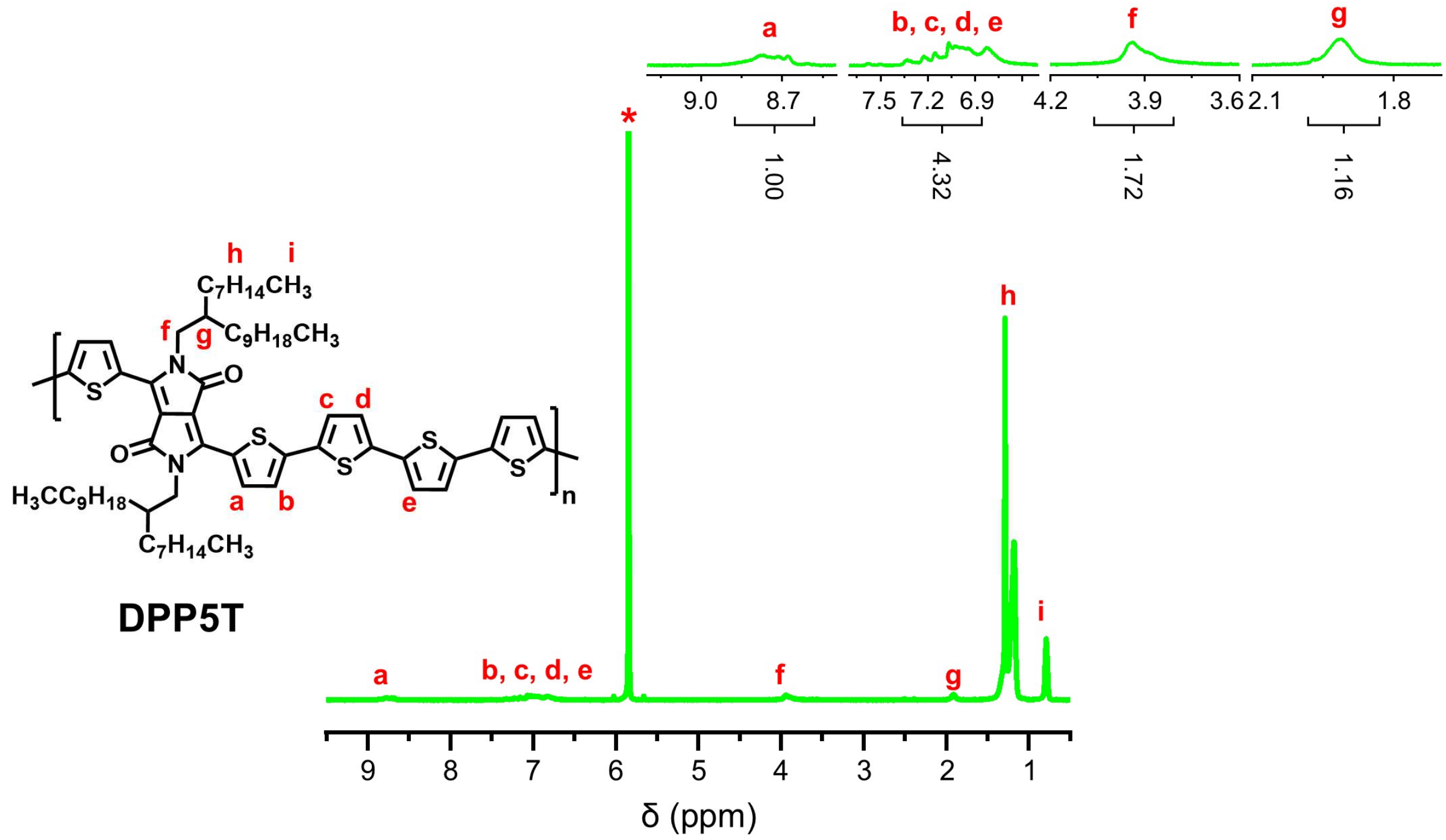

Figure S3. ^{1}H NMR spectra of (a) DPP3T, (b) DPPTTT, (c) DPP4T, and (d) DPP5T in $C_2D_2Cl_4$ (*), respectively.

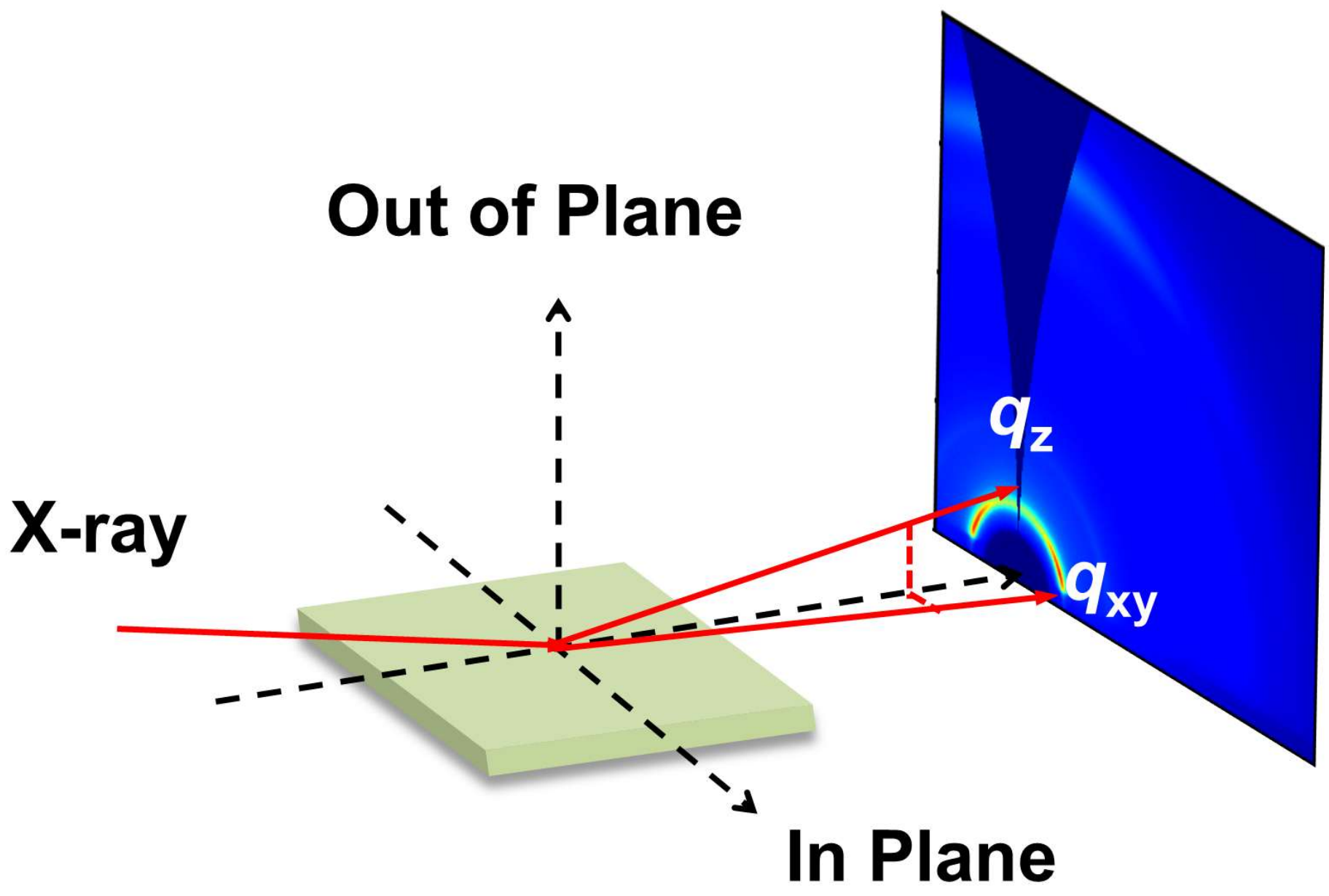

Figure S4. Schematic illustration of 2D-GIWAXS measurement on DPP samples.

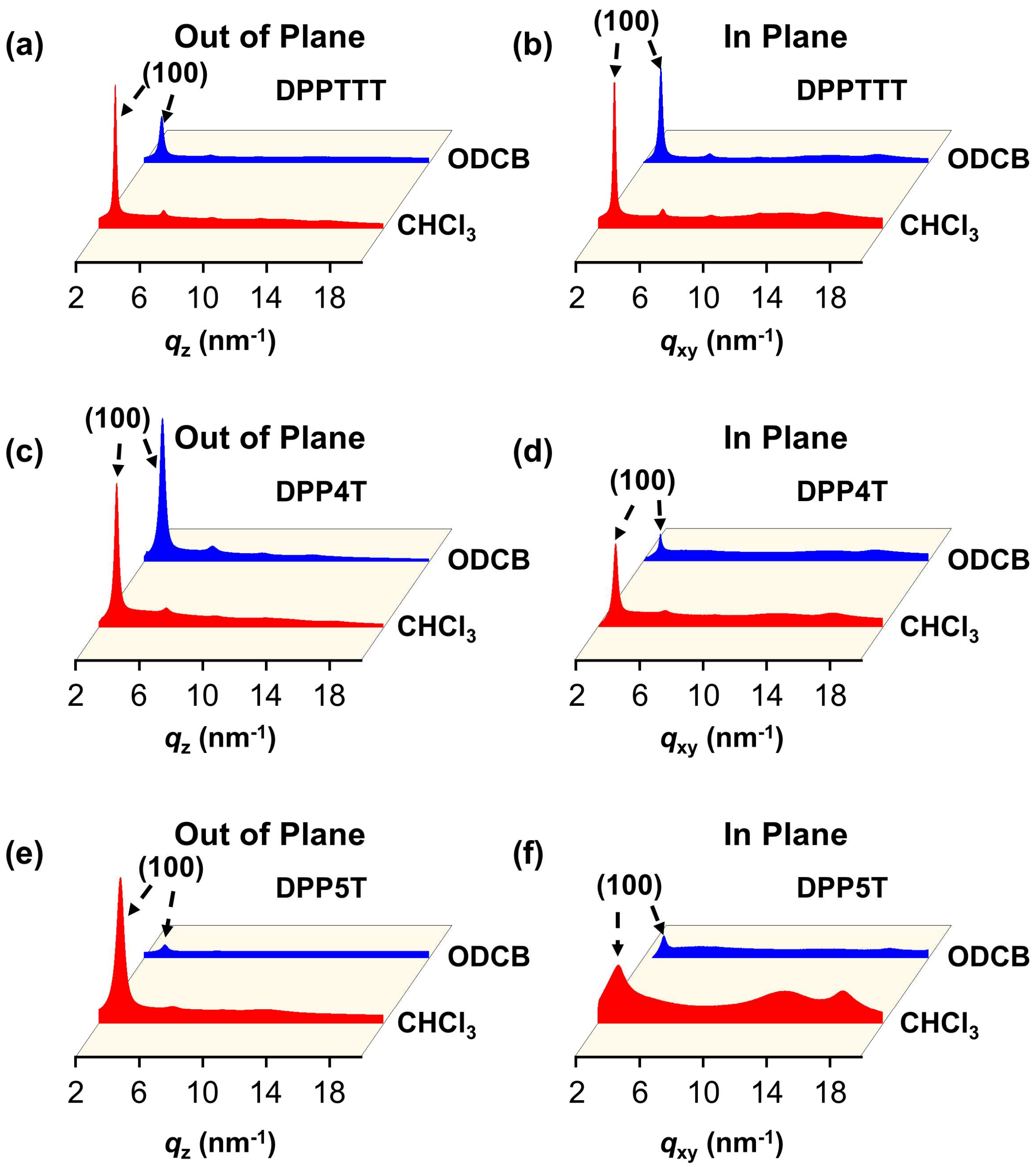

Figure S5. 1D-GIWAXS profiles of (a, b) DPPTTT, (c, d) DPP4T, and (e, f) DPP5T films obtained from the corresponding 2D-GIWAXS images along the (a, c, e) out-of-plane (q_z) and (b, d, f) in-plane (q_{xy}) directions, respectively.

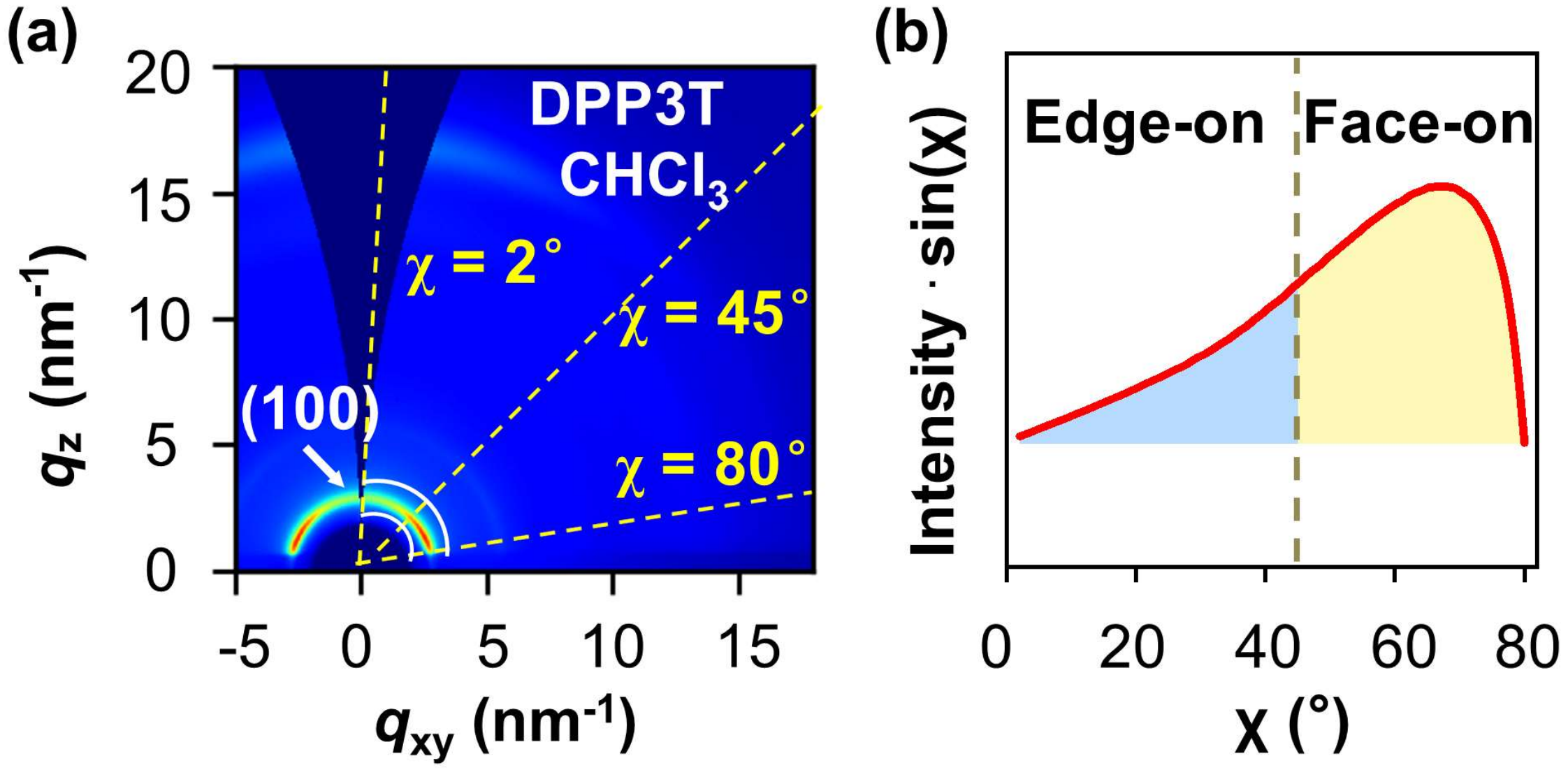

Figure S6. (a) Illustration of extracting the intensity of the DPP3T (100) peak from the 2D-GIWAXS image in Ewald sphere correction as a function of χ. The peak area is divided into χ = 2–45° and 45–80° regions, corresponding to the edge-on and face-on crystals, respectively. (b) Pole figure of the (100) peak of DPP3T thin film produced from $CHCl_3$. The peak intensity was multiplied by a geometric factor (sin(χ)) for Ewald sphere correction. The blue and yellow area represent the edge-on and face-on regions, respectively.

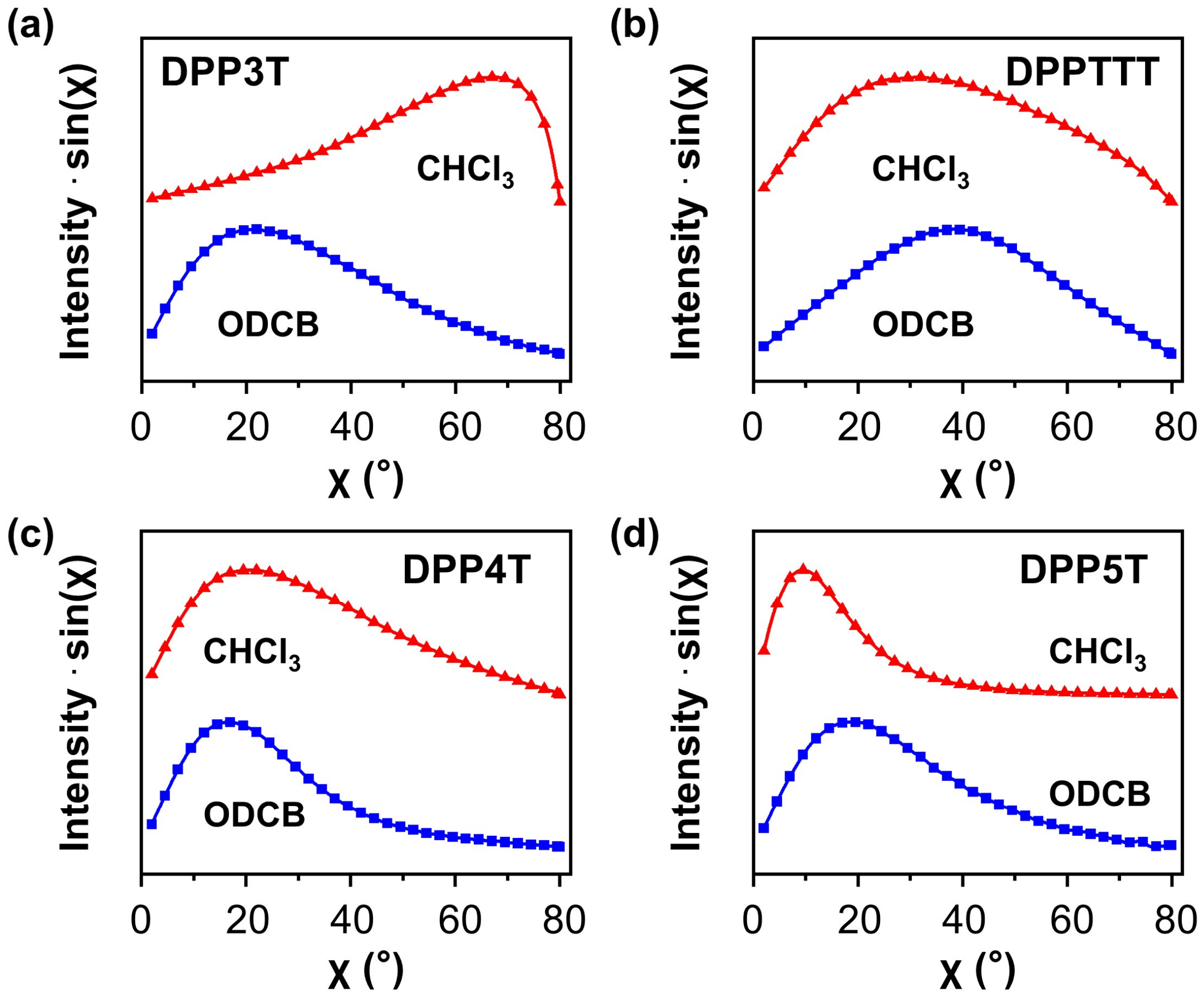

Figure S7. Pole figures of the (100) diffraction peaks extracted from the corresponding 2D-GIWAXS images of (a) DPP3T, (b) DPPTTT, (c) DPP4T, and (d) DPP5T thin films produced from $CHCl_3$ and ODCB, respectively. The peak intensity was multiplied by a geometric factor (sin(χ)) for Ewald sphere correction.

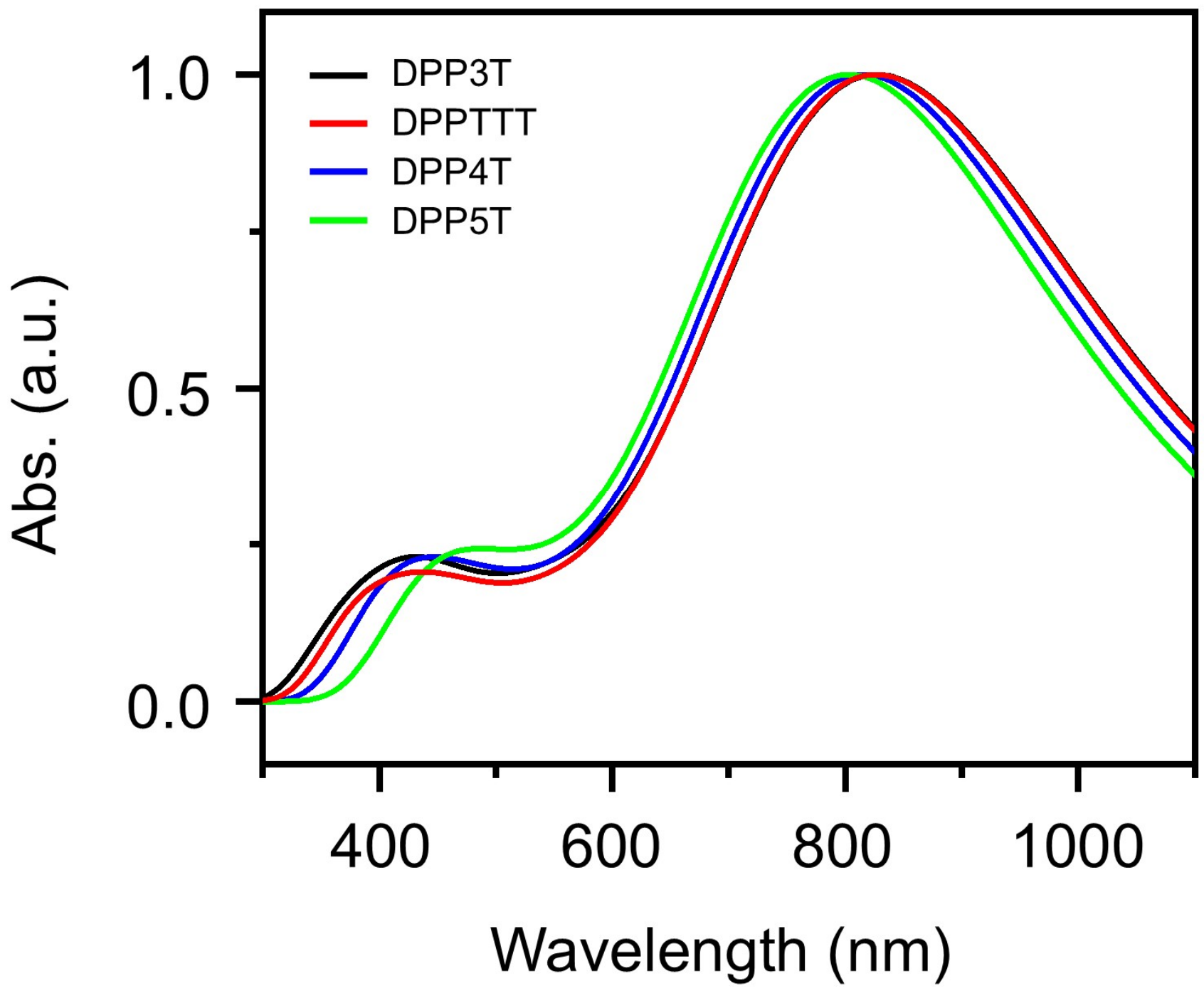

Figure S8. Calculated UV-vis absorption of DPP3T, DPPTTT, DPP4T, and DPP5T in $CHCl_3$ solution via PBE0/6-31G(d,p) basis set.

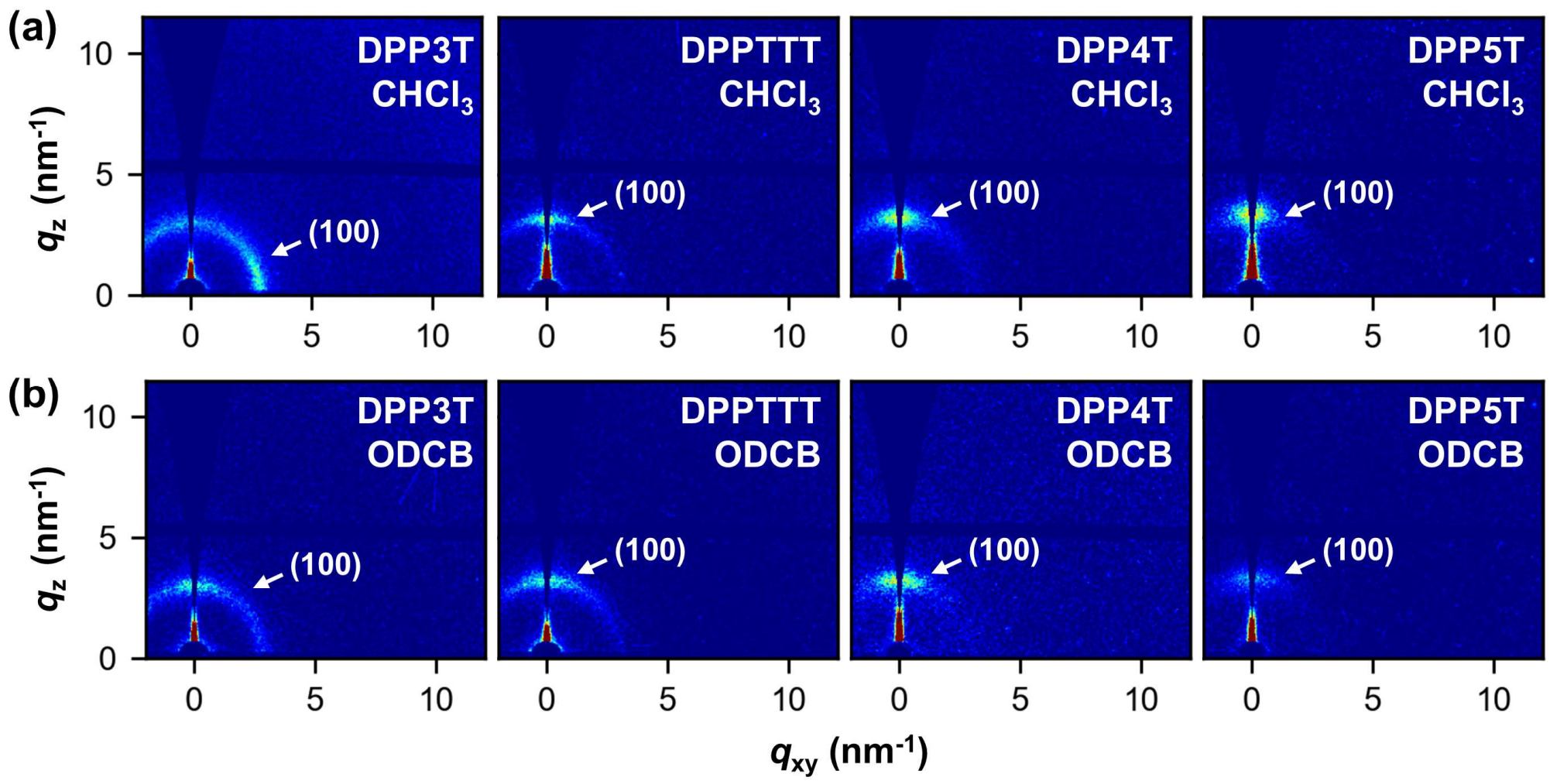

Figure S9. 2D-GIWAXS images of DPP3T, DPPTTT, DPP4T, and DPP5T thin film as-cast from (a) $CHCl_3$ and (b) ODCB at a lower concentration (0.2 mg/mL). The 2D-GIWAXS measurements were performed using a Xeuss 2.0 (Xenocs, France) equipped with a Cu anode (λ = 1.54 Å).

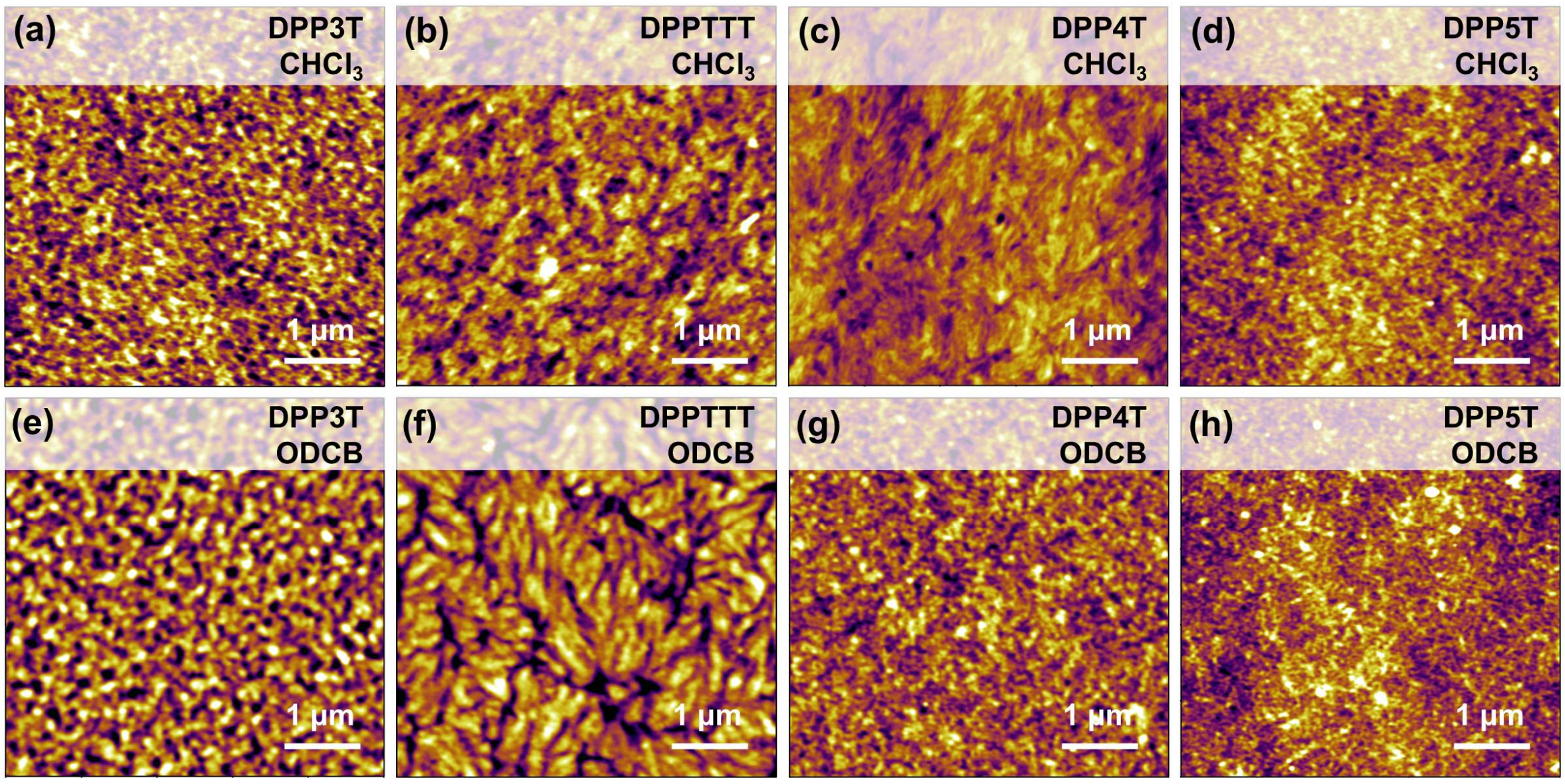

Figure S10. AFM images of four DPP thin films produced from (a–d) $CHCl_3$ and (e–h) ODCB.

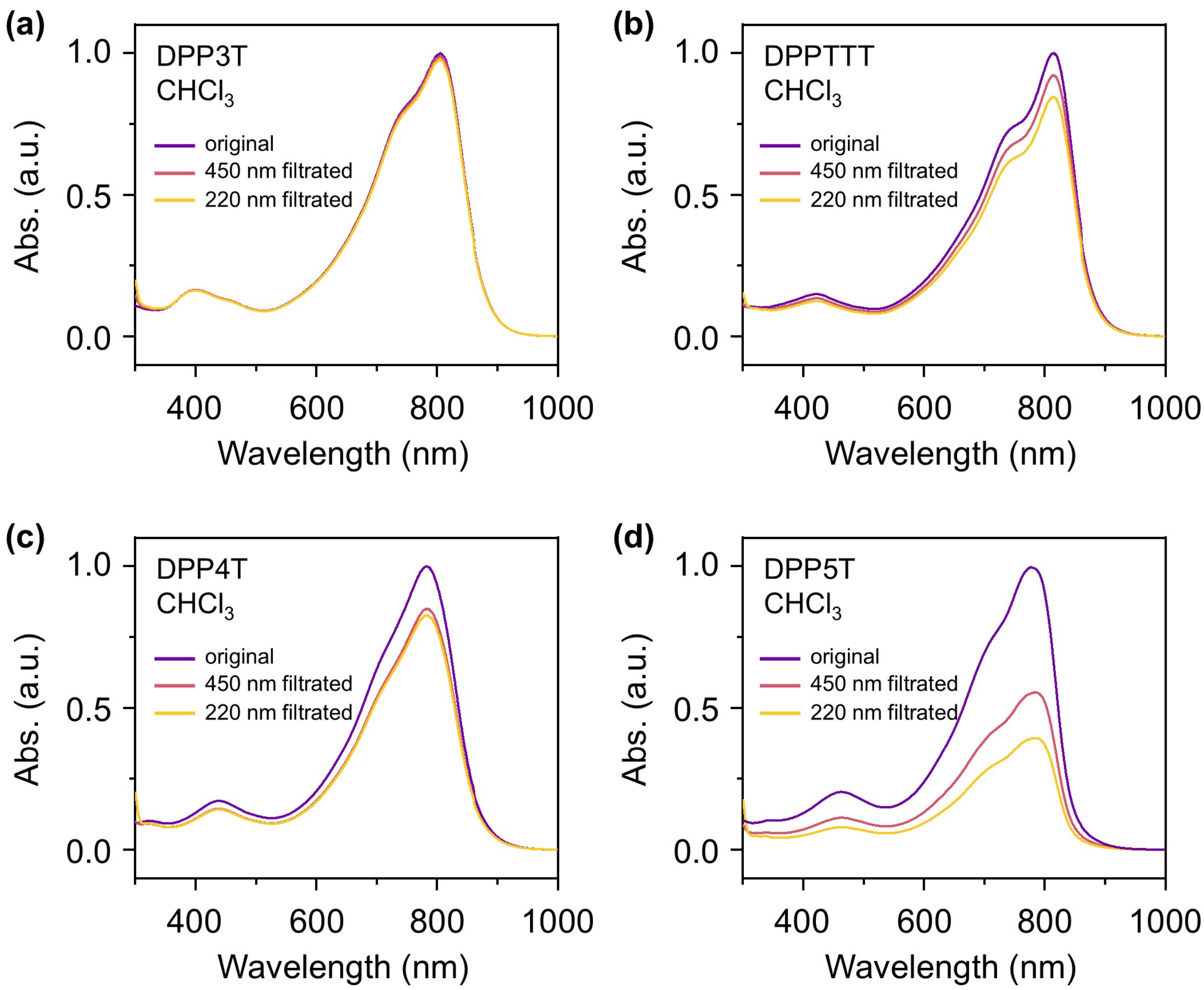

Figure S11. Absorption spectra in the filtration experiments of (a) DPP3T, (b) DPPTTT, (c) DPP4T, and (d) DPP5T in $CHCl_3$.

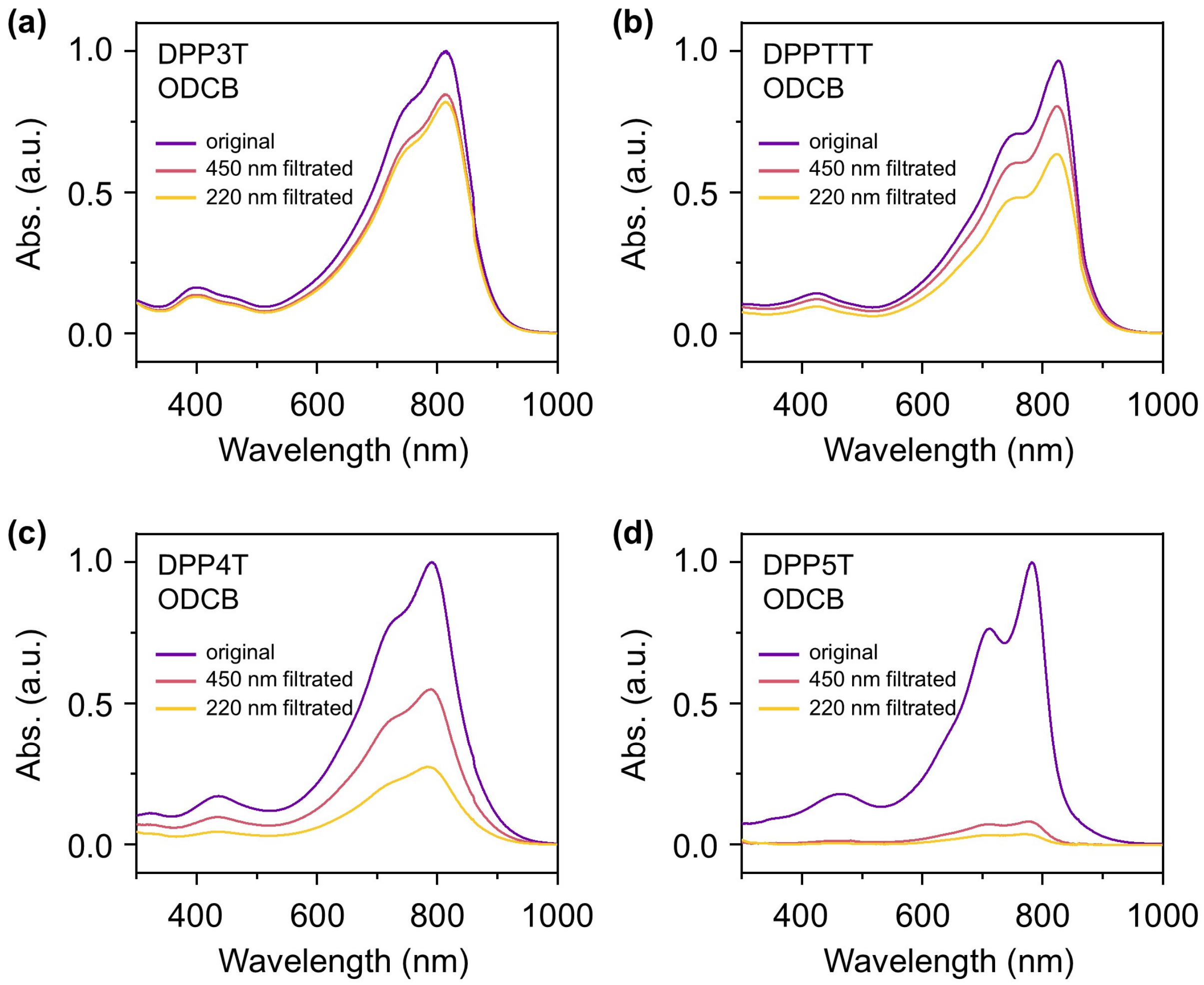

Figure S12. Absorption spectra in the filtration experiments of (a) DPP3T, (b) DPPTTT, (c) DPP4T, and (d) DPP5T in ODCB.

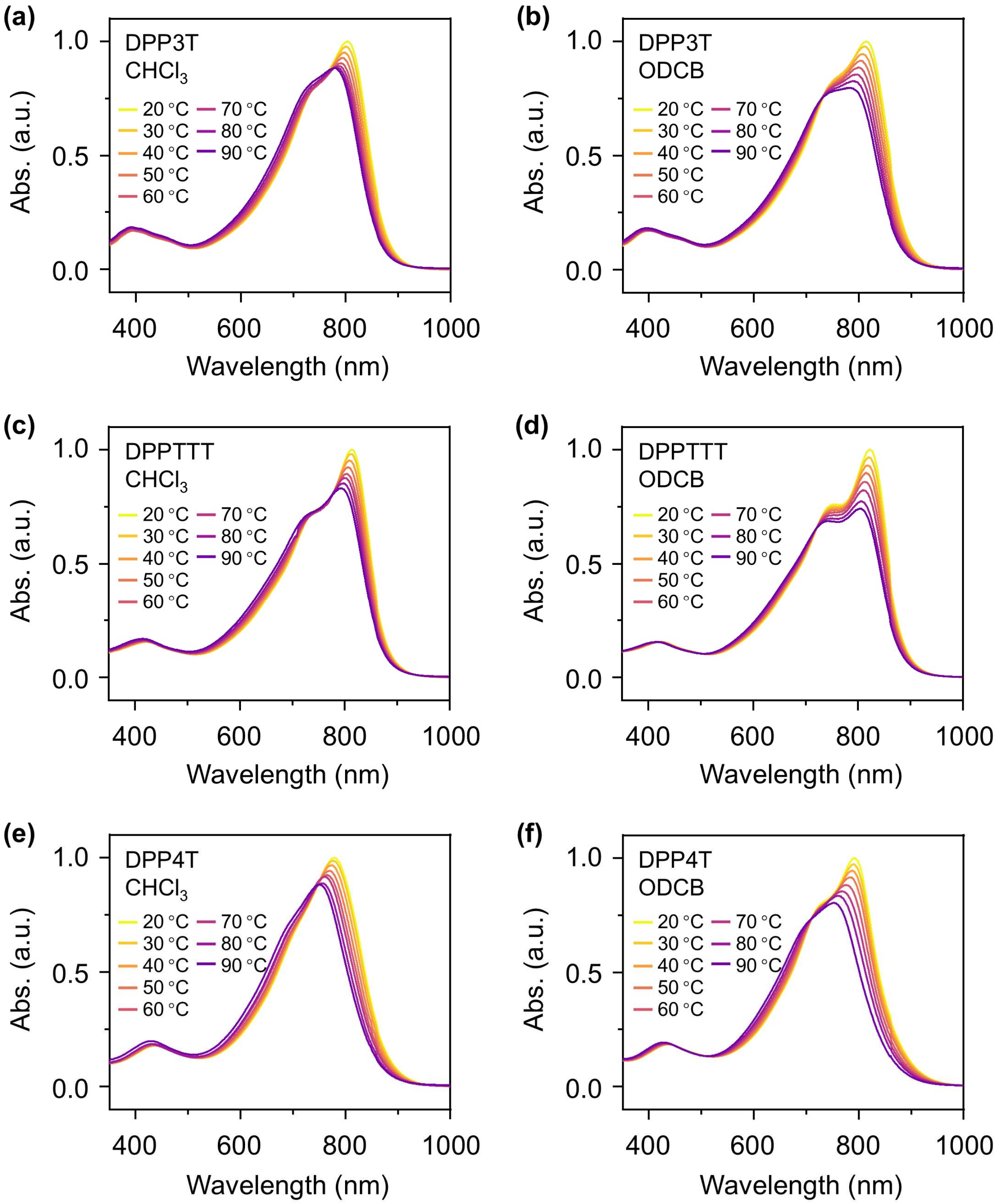
(a)
DPP3T
CHCl$_3$
(b)
DPP3T
ODCB
(c)
DPPTTT
CHCl$_3$
(d)
DPPTTT
ODCB
(e)
DPP4T
CHCl$_3$
(f)
DPP4T
ODCB
20 °C
30 °C
40 °C
50 °C
60 °C
70 °C
80 °C
90 °C
Abs. (a.u.)
Wavelength (nm)
0.0
0.5
1.0
400
600
800
1000

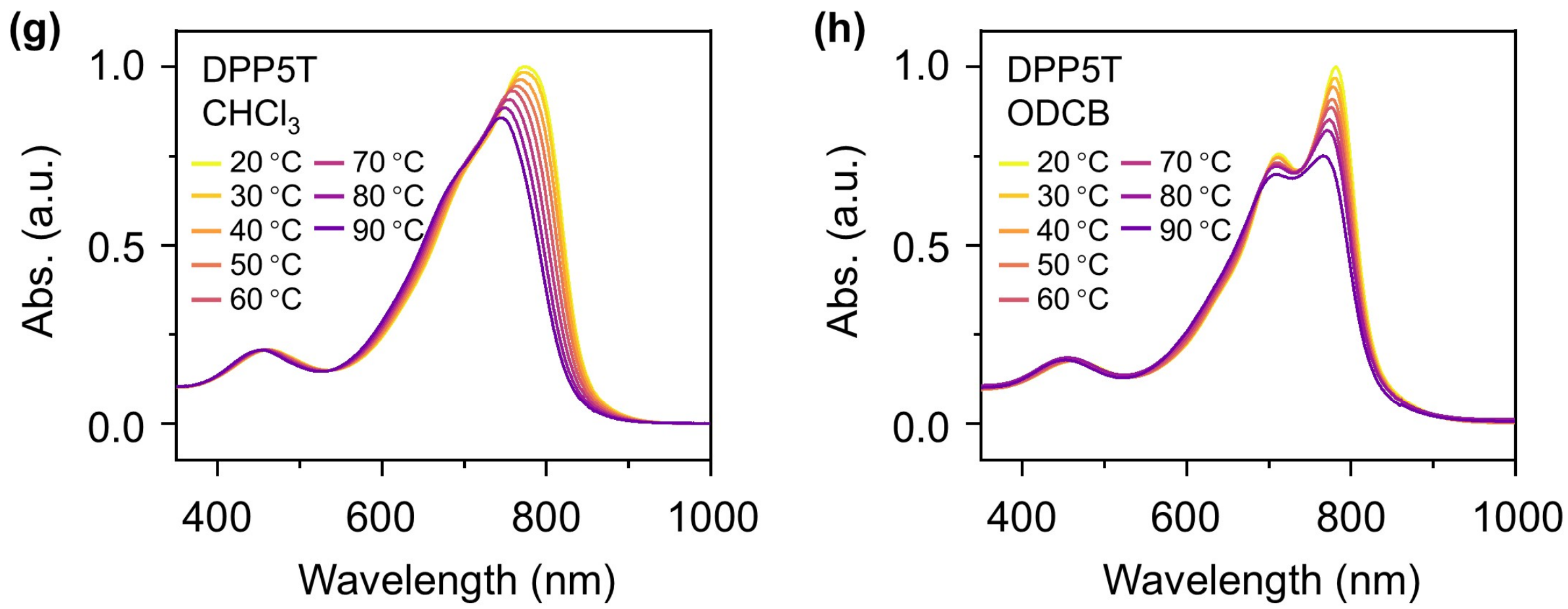

Figure S13. Temperature-dependent absorption spectra of (a, b) DPP3T, (c, d) DPPTTT, (e, f) DPP4T, and (g, h) DPP5T in (a, c, e, g) $CHCl_3$ and (b, d, f, h) ODCB, respectively.

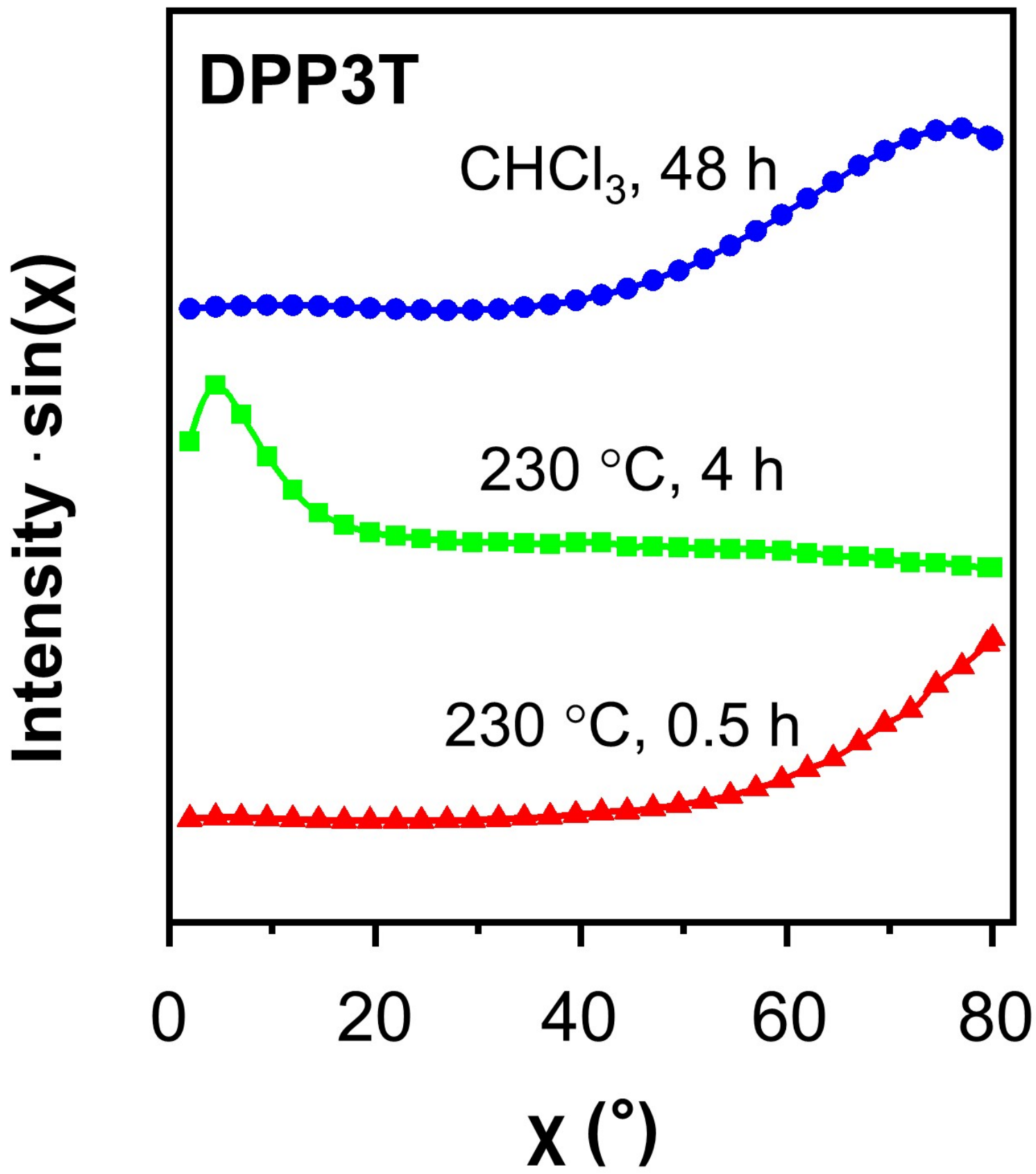

Figure S14. Pole figures of the (100) diffraction peaks extracted from the 2D-GIWAXS images of DPP3T films produced from $CHCl_3$ followed by annealing at 230 °C for 0.5 h, annealing at 230 °C for 4 h, and then annealing by $CHCl_3$ vapor for 48 h in sequence. The peak intensity was multiplied by a geometric factor (sin(χ)) for Ewald sphere correction.

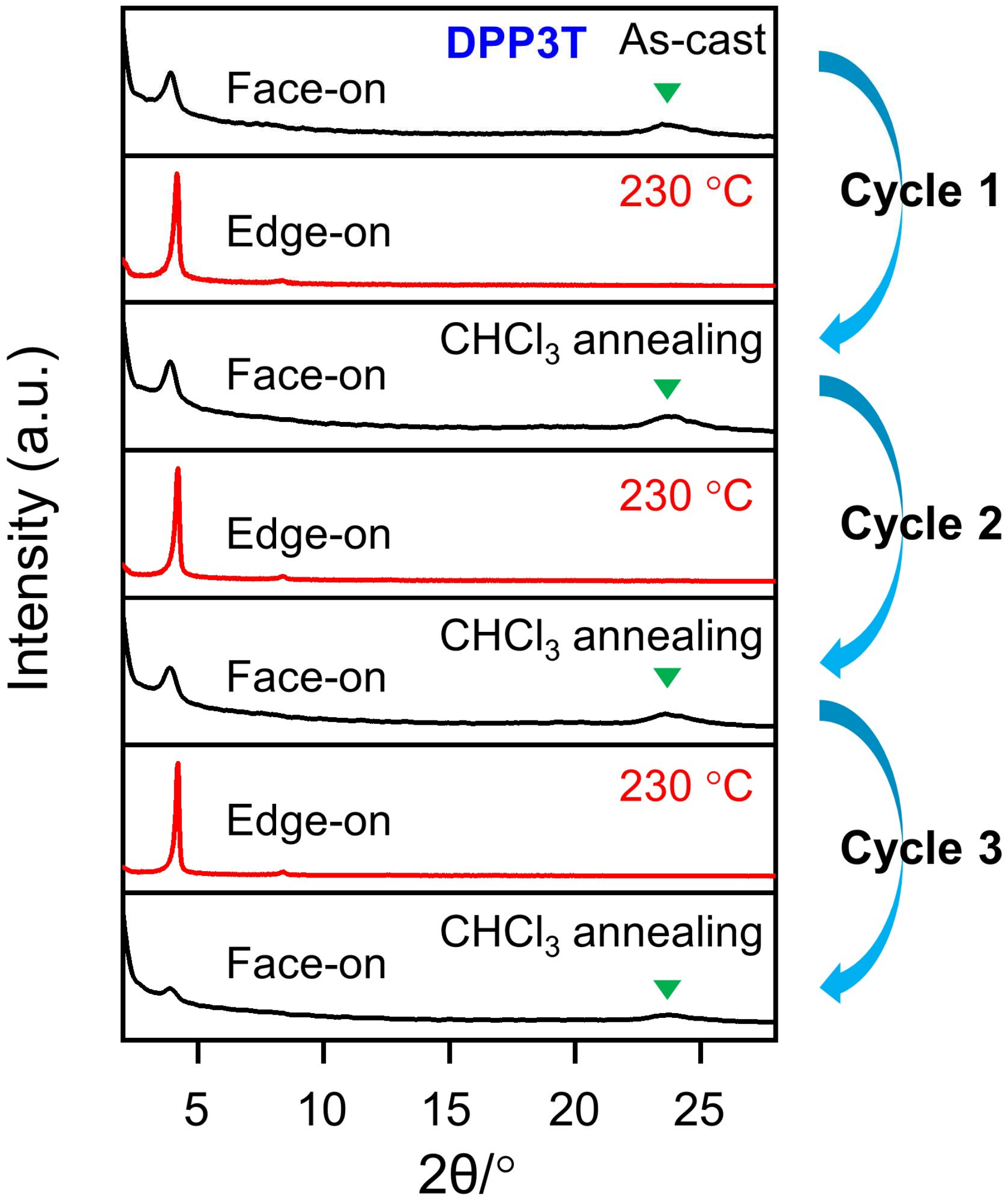

Figure S15. XRD spectra showing the reversible crystal orientation between face-on and edge-on in DPP3T thin films by alternating thermal and $CHCl_3$ vapor annealing. The measurement started from the DPP3T thin film produced from $CHCl_3$, exhibiting face-on orientation.

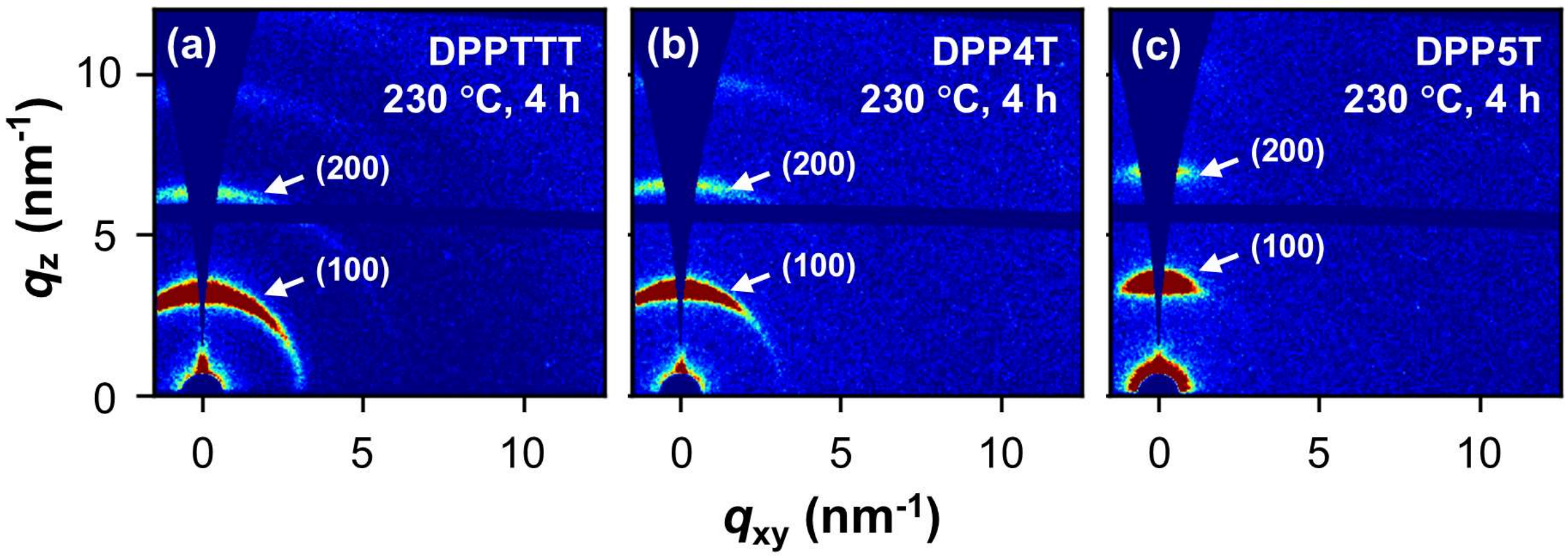

Figure S16. 2D-GIWAXS images of (a) DPPTTT, (b) DPP4T, and (c) DPP5T thin film as-cast from $CHCl_3$ and followed by annealing at 230 °C for 4 h, respectively.

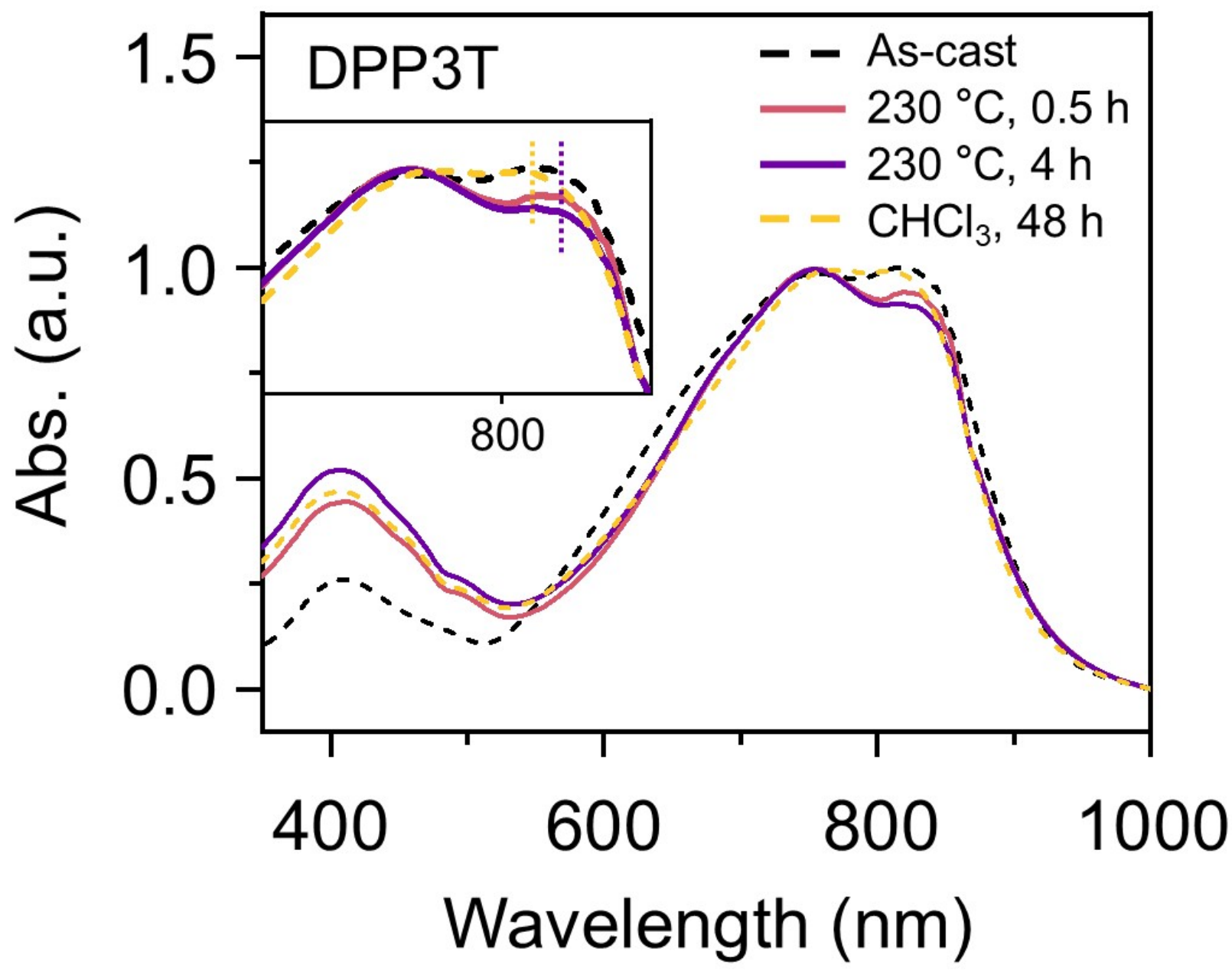

Figure S17. UV–vis absorption spectra of DPP3T thin film as-cast from $CHCl_3$, and followed by annealing at 230 °C for 0.5 and 4 h, and then annealed by $CHCl_3$ vapor for 48 h in sequence.

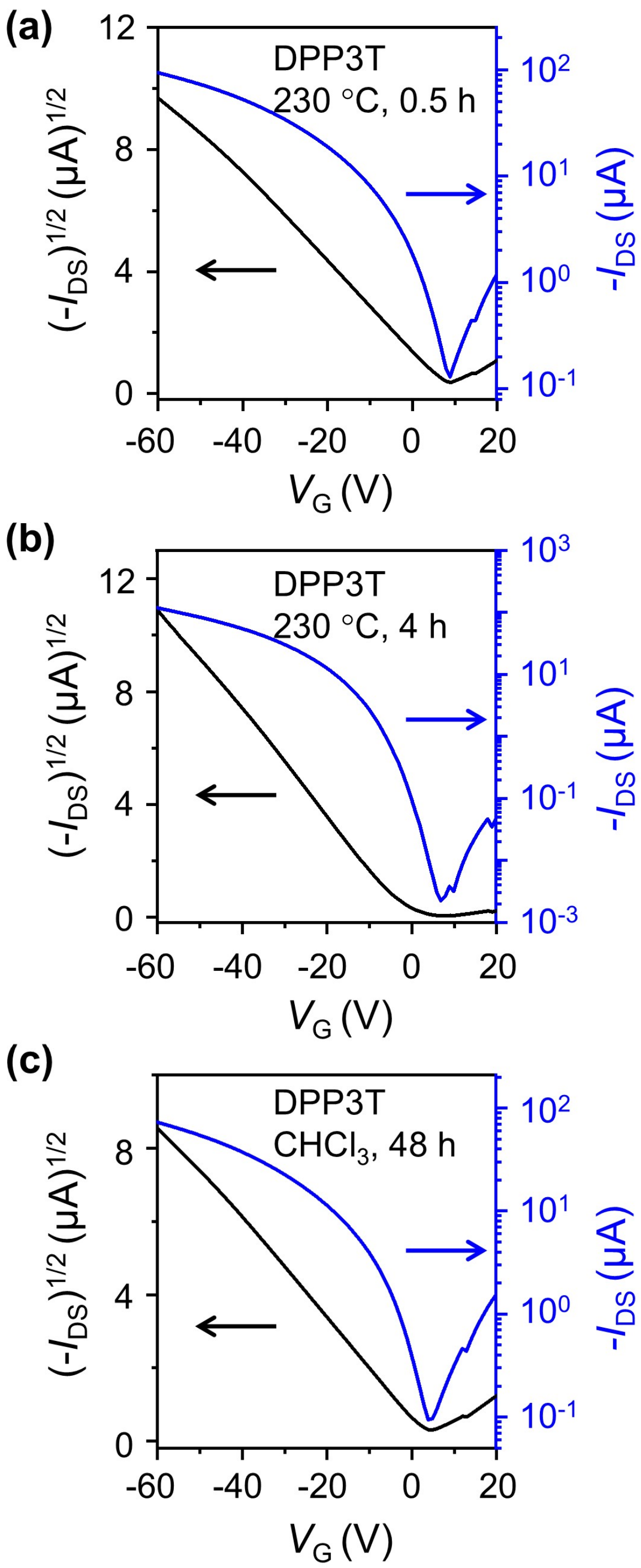

Figure S18. (a-c) Transfer curves of OFETs fabricated with DPP3T thin films produced from $CHCl_3$, followed by (a) annealing at 230 °C for 0.5 h, (b) annealing at 230 °C for 4 h, and then (c) $CHCl_3$ vapor annealing for 48 h in sequence.

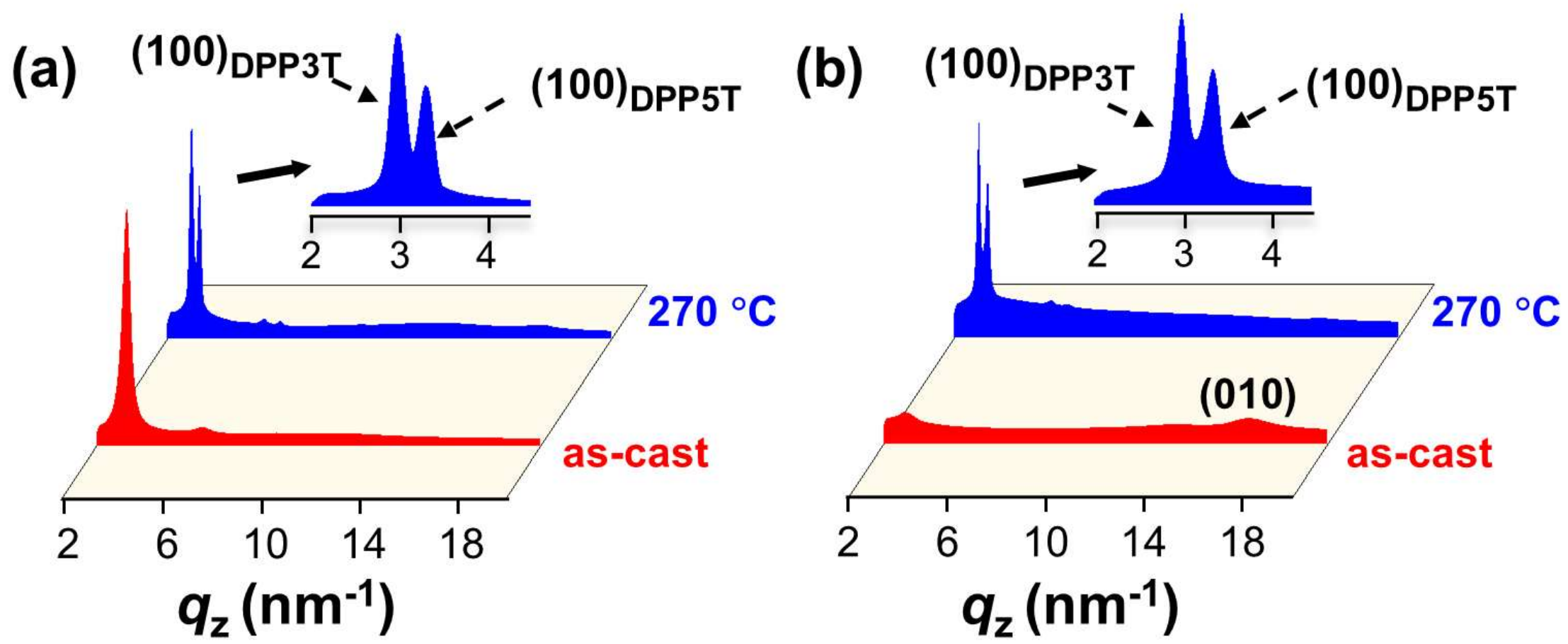

Figure S19. 1D-GIWAXS profiles of DPP3T/DPP5T blend at the weight ratios of (a) 1:1 and (b) 8:1 under different conditions (produced from $CHCl_3$, followed by thermal annealing at 270 °C, respectively).

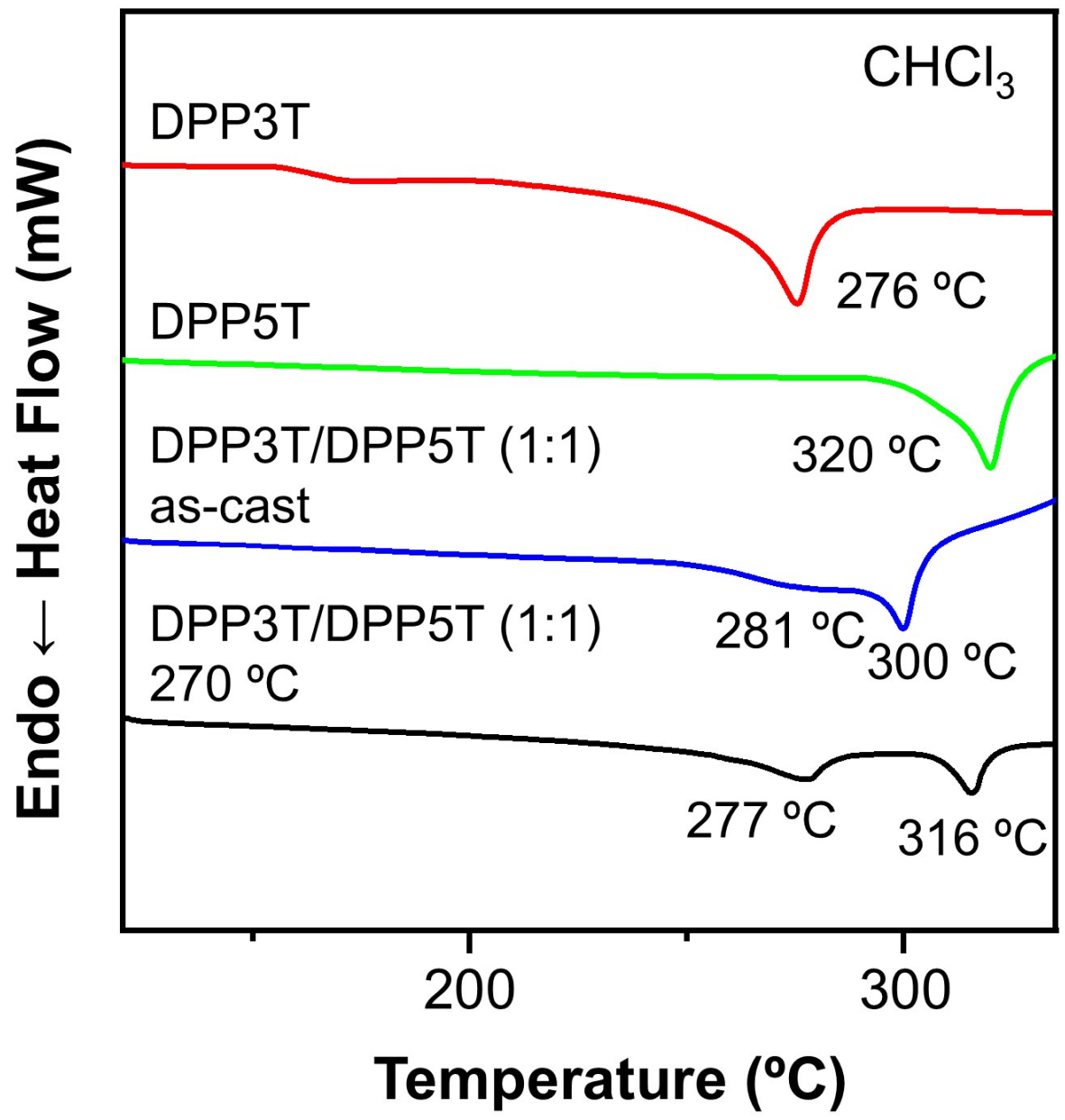

Figure S20. DSC curves of the as-cast DPP3T and DPP5T, and DPP3T/DPP5T blend (1:1) in the as-cast state and followed by thermal annealing at 270 °C.

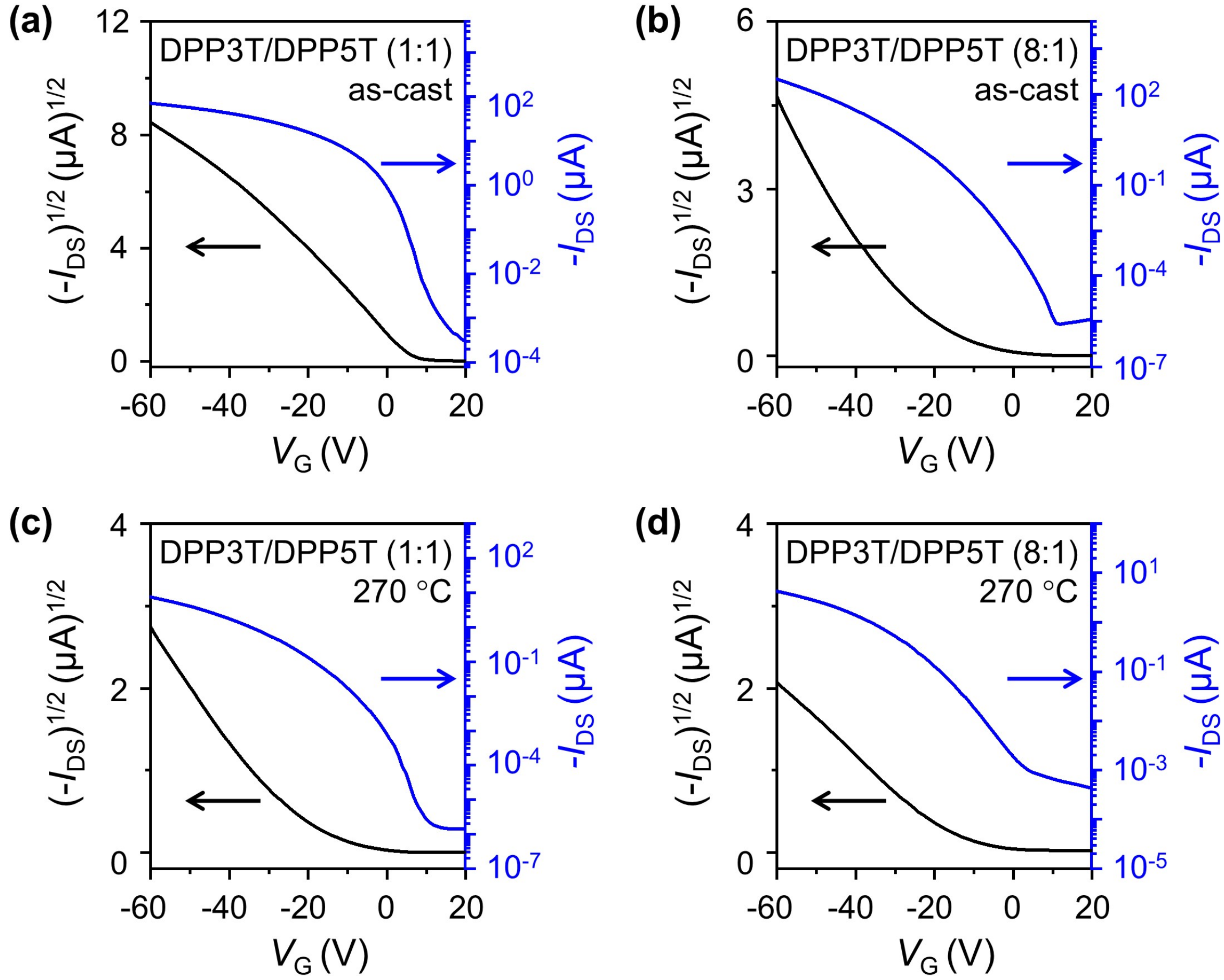

Figure S21. (a-d) Transfer curves of OFETs fabricated with (a, c) DPP3T/DPP5T blends (1:1) and (b, d) DPP3T/DPP5T blends (8:1) produced from (a, b) $CHCl_3$ solution, followed by (c, d) annealing at 270 °C.
